# In vitro model assesses the susceptibility of polymeric scaffolds for material-driven heart valve regeneration to calcification

**DOI:** 10.1007/s44164-025-00090-x

**Published:** 2025-07-15

**Authors:** Dewy C. van der Valk, Charlotte M. Hoes, Yunia M. H. Rasenberg, Paul A. A. Bartels, Livia Angeloni, Bente J. de Kort, Paul J. F. M. Janssen, Frank P. T. Baaijens, Anthal I. P. M. Smits, Carlijn V. C. Bouten

**Affiliations:** 1 Department of Biomedical Engineering, Eindhoven University of Technology, Groene Loper Building 15 (Gemini-Zuid), De Rondom 70 5612AP, Eindhoven, The Netherlands; 2 Institute for Complex Molecular Systems (ICMS), Eindhoven University of Technology, Ceres 1.31, Building number 7, De Zaale, Eindhoven, 5612 AJ The Netherlands; 3 Department of Mechanical Engineering, Eindhoven University of Technology, Traverse, PO Box 513, Eindhoven, 5600 MB The Netherlands

**Keywords:** Tissue regeneration, Mineralization, Scaffolds, 3D in vitro models, Cell-material interactions, Extracellular matrix

## Abstract

**Purpose:**

Material driven in situ heart valve tissue engineering (HVTE) prospects an alternative to non-living replacements. HVTE exploits bioresorbable (synthetic) scaffolds that guide neo-tissue formation. Proper scaffold design assesses and mitigates potential material-related risks, such as calcific nodule formation. Herein, we establish an in vitro model to investigate the calcification risk of materials for HVTE.

**Methods:**

Calcification was studied by culturing 3D scaffolds with porcine valvular interstitial cells in a phosphate-enhanced calcification medium (CM) for 3 weeks. The model was applied by testing three electrospun polymeric Tissue engineering (TE) scaffolds (PCL, PCL-BU, and PC-BU) against a bovine pericardial patch control. Additionally, the model included a 10% cyclic strain environment to evaluate hemodynamic effects.

**Results:**

TE constructs showed significantly less calcification compared to the pericardial tissue control, mirroring in vivo animal model findings. No differences in calcification were observed among the TE constructs, and cyclic strain did not affect calcification.

**Conclusion:**

The 3D in vitro model established in this study effectively mimics calcification in TE material constructs, aiding in systematic testing and comparison of cardiovascular TE materials. It can help understand calcification principles and evaluate potential risk factors (e.g., strain). As such, the model will support the design of biomaterials for in situ HVTE in particular and implantable polymer grafts in general.

**Supplementary Information:**

The online version contains supplementary material available at 10.1007/s44164-025-00090-x.

## Introduction

Deaths from calcific aortic heart valve disease (CAVD) have continued to rise over the past 20 years [1]. During the disease, valvular interstitial cells (VICs) in the tissue change from a quiescent to an osteoblastic phenotype due to oxidative stress, lipoproteins, inflammatory macrophages, high phosphate levels, or loss of mineral-deposition inhibition [2]. This leads to calcified vesicles, microcalcifications, and macrocalcifications. Unlike bone mineralization, valvular calcification particles are disorganized and isolated from collagen fibers [3]. Currently, there is no medical treatment for CAVD, necessitating surgical valve replacement in the end stage of the disease when valves become severely stenotic. The most common replacement valves are bioprosthetic valves, followed by mechanical prostheses, recommended in patients below 50 years of age [4–6]. Mechanical valves require lifelong anticoagulation therapy, while bioprosthetic valves often suffer from structural degeneration and calcification, necessitating re-operation [7].

New valvular replacements are being developed for CAVD, rheumatic heart valve disease in low- and middle-income countries [8], and congenital heart disease in children [9]. In situ heart valve tissue engineering (HVTE) offers off-the-shelf strategies to grow living valves inside the body, adapting to the patient’s changing hemodynamic environment without anticoagulation therapy [10]. HVTE utilizes the host’s inflammatory response to degrade an implanted carrier material—or scaffold—while inducing new tissue formation, gradually replacing the scaffold with autologous graft tissue [11, 12]. HVTE scaffolds may provide a more durable treatment option than current bioprosthetic valves [4]. The advantageous prospects of synthetic in situ HVTE have given rise to various animal studies using different materials [13–17], as well as initial clinical trials for pediatric purposes [18].

With increasing interest for in situ HVTE, evaluating valvular scaffolds for safety and effectiveness in treating valvular diseases like CAVD is crucial, but methods to do so are virtually missing. Recently, we systematically assessed calcification in HVTE materials used to replace the pulmonary valve in large animal models. We found that 35% of evaluated animals showed some degree of (most micro-) calcification, with 33% in animals receiving synthetic scaffolds for in situ HVTE [19]. Performing extensive animal studies for scaffold screening and optimization is far from ideal and does not allow for systematic, real-time analysis of the mechanisms of scaffold material calcification and consequent scaffold optimization. Higher-throughput in vitro models can aid in screening materials, understanding undesirable remodeling effects, and optimizing new material fabrication. Currently, 2D and 3D in vitro models study VIC-like responses within CAVD [20, 21] and cellular responses within HVTE materials [22], but models studying VIC-related calcification within HVTE materials are lacking.

Here, we propose a versatile in vitro model to test and better understand calcification of synthetic polymer-based valve scaffolds for in situ HVTE. Important hallmarks for such a model include (1) use of 3D polymer scaffolds that mimic in situ tissue formation, (2) a relevant cell source, (3) a calcification-permitting medium and outcome parameters to capture the in vivo mechanisms, such as calcification and collagen formation, (4) a clinically relevant control material, and (5) the ability to capture relevant in vivo-like risk factors for calcification like hemodynamics (e.g., physiological strain).

We explored biodegradable materials poly-ε-caprolactone (PCL) and supramolecular elastomers bis-urea chain extended PCL (PCL-BU) and BU chain extended polycarbonate (PC-BU). PCL is widely used in vitro and has been associated with calcification in several vascular implant studies [23–26]. Our lab designed and tested PCL-BU and PC-BU vascular and valvular grafts for in situ tissue engineering [16, 27, 28]. These materials are often processed into 3D scaffolds via electrospinning, with fiber diameters around 4 µm optimal for cellular integration [22]. Mechanical characteristics such as stiffness and crystallinity were considered, as these factors can have a significant effect on VIC proliferation, differentiation, and calcification [20, 29].

Current clinical biological valve replacements mainly consist of glutaraldehyde-fixed bovine or porcine materials [30], which often require reoperation due to calcification [7]. Here, fixed bovine pericardial tissue, which is normally used to make biological valve implants, was used as a control against the synthetic materials.

Porcine valvular interstitial cells (pVICs) are a suitable alternative to human VICs due to their fast proliferation, ability to differentiate and form calcification, and the anatomical and physiological similarities between pig and human hearts [31]. Porras et al. developed a culture method to deactivate pVICs cultured on tissue culture polystyrene (TCPS) to a quiescent state [32], optimizing the cell source for studying cell activation and calcification after scaffold-cell interaction. To enable calcification without inducing medium-mediated diseased osteogenic cell differentiation, as a medium source, we opted only to use elevated levels of inorganic phosphate (β-glycerophosphate) only [33].

To summarize, in this study, we established an in vitro platform to study calcification in polymer scaffold materials used for in situ HVTE. To develop the model, we combined VICs, electrospun fibrous scaffold materials, a clinically relevant control material, and a calcification-permitting medium. After 21 days, we evaluated cell, extracellular matrix (ECM), and calcification differences. We applied the model to study the effect of material differences and cyclic strain on cardiovascular scaffold calcification. This model may be used in future studies to explore mechanisms underlying the calcification of materials for HVTE. This could aid the screening and optimization of polymer scaffolds to prevent this outcome for novel materials.

## Materials and methods

### Experimental outline

**Fig. 1:**
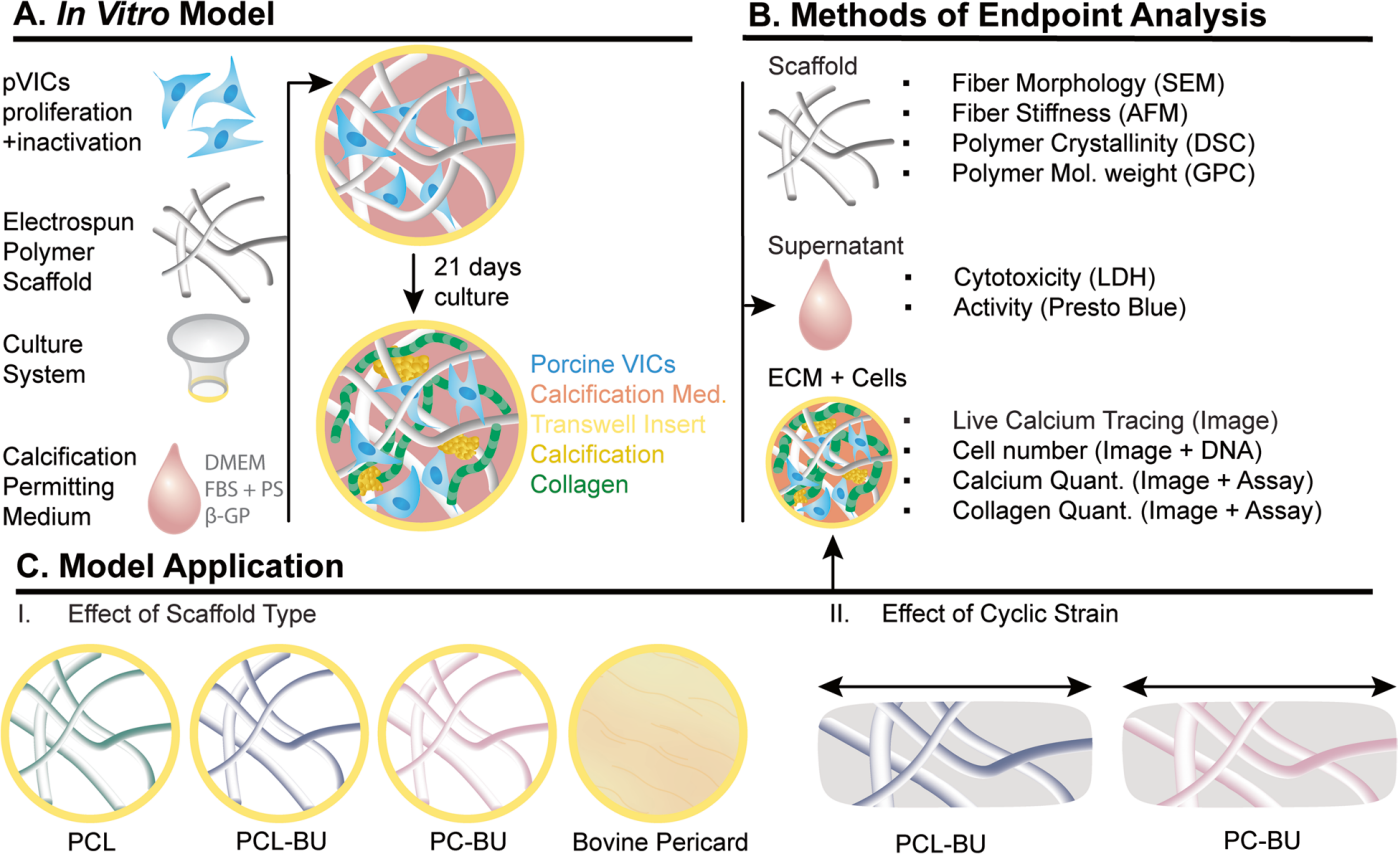
Study Design. **A** Within the in vitro model, pVICs are seeded on fibrous, electrospun polymer scaffolds in Transwell inserts and cultured for 21 days in calcification-permitting medium (CM). **B** Cultured constructs are evaluated for cytotoxicity, calcification, and collagen formation after 3 weeks of culture. **C** The application of the model has been tested in two ways: (I) three materials used for in situ tissue engineering are selected and thoroughly analyzed before cell seeding and after cell culture. They are compared to a clinically used material, glutaraldehyde treated bovine pericardium. (II) An applicational pilot evaluated the calcification potential of constructs cultured under cyclic stretch. pVICS, porcine valvular interstitial cells; DMEM, Dulbecco’s modified eagle medium; FBS, heat-inactivated fetal bovine serum; PS, penicillin/streptomycin; β-GP, β-glycerophosphate; SEM, scanning electron microscopy; AFM, atomic force microscopy; DSC, differential scanning calorimetry; GPC, gel permeation chromatography; LDH, lactate dehydrogenase; IF, immunofluorescence; PCL, polycaprolactone; PCL-BU, bisurea chain extended PCL; PC-BU, bisurea chain extended polycarbonate

To build the in vitro model for the evaluation of calcification in HVTE materials, first, after proliferation (and activation) on tissue culture polystyrene (TCPS), pVICs were made quiescent using a fibroblast inactivation medium (Fig. 1A). Then, quiescent pVICs were seeded on scaffolds that were suspended in medium using sterile Transwell inserts. To induce and investigate calcification within realistic culture times while not inducing cell differentiation, a calcification permitting medium (CM) was used to culture cell/scaffold constructs for 3 weeks after cell seeding. Constructs, supernatant, and ECM with cells were evaluated before, during, and after culture (Fig. 1B). Before cell seeding, the similarity between scaffolds of different production batches was assessed. During cell culture, cell cytotoxicity and calcification were evaluated in real time. After 3 weeks of culture, calcification, collagen formation, cell proliferation, and viability were assessed and quantified.

The model was applied to study factors possibly influencing HVTE calcification. First, three types of polymeric materials (PCL, PCL-BU, and PC-BU) were compared to pericardial tissue that is used to make clinical biological valve implants on their propensity to calcify (Fig. 1C(I).). Secondly, since hemodynamics also play a critical role in the TE process and the calcification of native valves [34], a pilot study was established to study the effect of cyclic strain on material scaffold calcification within this 3D model (Fig. 1C(II)).

### Scaffold preparation

One batch of raw PCL polymer material (Purasorb, PC-12) was purchased from Corbion (Gorinchem, the Netherlands), and multiple batches of raw PCL-BU and PC-BU materials were supplied by Symochem (Eindhoven, the Netherlands). Polymers were diluted into working polymer solutions containing chloroform (CHCl_3_, ethanol stabilized, Sigma, 288,306) and methanol (MeOH, VWR Chemicals, 20,903.368) or hexafluoro-isopropanol (HFIP,- Sigma) according to Table 1. Working polymer solutions were electrospun into fibrous scaffold sheets within a climate-controlled electrospinning cabinet (Vivolta, Waalre, the Netherlands). Using a regulated flow, the polymer solution was extruded from a positively charged moving nozzle (translation speed 12 mm/s) and deposited on a negatively charged rotating collector (303 rpm for a 2 cm collector or a relative tangential speed). Electrospinning parameters were optimized to get randomly oriented fibers of approximately 4 µm thickness to allow for good cell infiltration into the scaffold [22]. Since experiments were performed using several different material polymer batches, the optimization and settings were slightly different for each experiment. Final settings per polymer are summarized in Table 1.

**Table 1:** Polymer solution preparation and electrospinning settings to produce comparable scaffold sheets

		Material		
		PCL	PCL-BU	PC-BU
Polymer solutions	% polymer (wt% or w/v)	30% w/v	18% wt%	16% wt%
	Solvent (w/w)	CHCL_3_:MeOH (90:10)	CHCL_3_:MeOH (98:2)	CHCL_3_:MeOH (85:15)
Electro-spinning parameters	Temperature (°C)/humidity (%)	25/25	23/30	23/50
	Nozzle-collector distance (cm)	20	18	12
	Nozzle voltage (kV)	+ 18	+ 17 + →18.5 (ramp over time)	22
	Collector voltage (kV)	− 1	− 1	0
	Flow rate (µl/min)	21	28	40
	Gas shield flow	0	200	600

Scaffolds were spun until a thickness of approximately 300 µm. Scaffold thickness was measured using built-in laser thickness measurement (Vivolta). After spinning, scaffold sheets were cut from the collector and dried in a vacuum chamber at room temperature (RT) overnight. They were stored at RT (first experiment) or at − 20 °C (experiments 2–4) until further processing or experimentation. Supplementary Table 1 shows the electrospun scaffolds used for each experiment.

Bovine pericardial patch tissue (500 µm) was purchased from Edwards Lifescience (Model 4700, Breda, the Netherlands) to be used as a clinical control tissue. The pericardial patch control was left in glutaraldehyde and stored at 4 °C until use.

### Cell expansion

#### Porcine valvular interstitial cells (pVICs)

Primary pVICs were isolated from four individual male pig aortic valve leaflets using collagenase digestion as previously described [32]. pVICs were expanded in pVIC growth medium (p-GM) consisting of low glucose DMEM (D6046, Sigma Aldrich), supplied with 10% FBS and 1% P/S. Medium was changed every 2–3 days, and cells were passaged at 90% confluency, up until passage 4. Then, pVICs were transferred to collagen-coated flasks. After 24 h of further culture, the p-GM medium was changed to fibroblast inactivation medium (FIB), consisting of low glucose DMEM with 2% heat-inactivated FBS, 1% P/S, 5 µg/ml insulin, and 10 ng/ml fibroblast growth factor 2 [FGF-2; PeproTech] to achieve cell quiescence, as described before [32]. After 1 week of culture in FIB medium, quiescent pVICs were transferred to scaffolds for experimental culture. To evaluate the effectiveness of FIB culture, one set of pVICs (donor4) was cultured in FIB on collagen-coated coverslips, fixed in formalin, and compared to pVICs cultured in normal medium (NM) after 7 days of culture. Supplementary Table 1 shows the distribution of animal donors per experiment.

### Scaffold sterilization, cell seeding, and culture

#### Static pVIC culture

A 6.5 mm Transwell inserts without membrane (CLS3472, Corning, Merck, Darmstadt, Germany), custom-made fitting peek-rings, and polypropylene spacer rings were washed in virkon S for at least 24 h, flushed with demineralized water, and washed for at least 24 h in 70% ethanol prior to preparation. An 8 mm diameter circles were punched out of the polymer scaffolds. Polymer scaffolds, inserts, peek-rings, and spacer rings were then UV-sterilized 3 times for 5 min while flipping them in between rounds. Pericardial patch tissue was punched out into 8 mm diameter circles under sterile conditions and washed 3 times in phosphate buffered saline (PBS). Scaffold and pericardial circles were then placed in a peek ring, and an insert was clicked on top, securing the scaffolds and pericardial circles in place. Then, the whole system was again UV sterilized 3 times for 5 min. After this, the inserts were placed in a 24-well plate, using the spacer rings between the top of the plate and the insert, to create enough space for medium below the insert, hydrating the scaffolds from both top and bottom sides. Growth medium was added at 1.5 ml to the system, and inserts were left in an incubator at 37 °C overnight.

The following day, medium was removed using a vacuum suction system, and scaffolds (not pericardial) were dried to make them more hydrophilic, thereby improving the distribution of cells throughout the whole scaffold when seeding. Cells were removed from their appropriate culture conditions, counted, and seeded on top of the dried scaffolds and (not dried) pericard at 0.5 × 10^6^ cells/scaffold (translating to approximately 1 × 10^6^ cells/cm^2^ surface area). After two rounds of cell seeding, with 15 min waiting time to achieve cell saturation of the scaffold, inserts were supplied with 1.5 ml growth medium and left overnight.

For normal medium (NM) groups, the medium was changed the day after seeding to DMEM, with 5% heat-inactivated FBS and 1% penicillin/streptomycin (P/S). For calcification medium (CM) groups, NM was supplemented with 10 mmol/L β-glycerophosphate (β-GP). All medium was changed every 2–3 days for 21 days of total culture.

#### Culture under strain

Rectangular scaffold strips (20 × 5 mm) were cut from the electrospun PCL-BU and PC-BU sheets. The outer edges of the strips were glued onto uncoated BioFlex culture plates (Flexcell®, Burlington, NC, USA) using Silastic MDX4-4210 (Dow Corning, MI, USA) with polymerization overnight at room temperature. The scaffold plates were then sterilized by washing with 30% ethanol, followed by two washes with sterile PBS, and then by 15 min UV sterilization. Scaffolds were then incubated overnight in 4 ml growth medium, after which the medium was removed, and pVICs were seeded as described above and left overnight in GM.

The following day, medium was changed to CM, and a subset of the cell-laden scaffold strips was subjected to cyclic strains of a 10% application magnitude at a frequency of 0.5Hz using the Flexcell FX-5000™ Tension System (Flexcell®, Burlington, NC, USA) for 21 days. The other subset of scaffolds was left unstrained (static control).

Strain distribution and maxima were characterized using digital imaging on graphite-coated scaffolds as described before, using the Green–Lagrange strain, a measure of deformation calculated from $\text{E}gl = 1/2 (\text{FT }\times \text{ F}-\text{I})$ with *F* as the deformation gradient tensor, *T* the transpose, and *I* the identity tensor [35]. Graphite-coated scaffolds were filmed and processed within Global Digital Image Correlation (GDIC) software from images at 60 frames/s [36].

### Outcome analysis

#### Cytotoxicity and cell viability in scaffolds to test material-induced cytotoxicity

To establish if materials induce cell death and if this could potentially influence calcification, cytotoxicity was tested during the experiment using the CyQuant™ lactate dehydrogenase (LDH) assay (C20301, ThermoFisher Scientific) according to the manufacturer’s protocol. Briefly, a positive control was created by incubating a sacrificed construct with 1:10 of lysis buffer. Fifty microliters of medium was taken from the control, scaffold culture groups, as well as a non-cell medium (negative) control. Samples were incubated for 30 min with LDH reaction mixture, after which the absorbance was read at 490 and 680 nm using a plate reader. Cytotoxicity was calculated as a percentage of 100% cytotoxicity using (Eq. 1):

1 $$\frac{scaffold\, measured\, absorbance\, \left(490-680\right)-Negative\, control\, (490-680)}{Positive\, control \,\left(490-680\right)-Negative\, control\, (490-680)}$$

Cellular activity was measured using the PrestoBlue™ assay (A13261, Invitrogen), according to the manufacturer’s protocol, only on day 21 of the experiment. Cells were incubated with 1:10 of PrestoBlue reagent in NM for 45 min, after which 100 µl of the PrestoBlue-contained NM was transferred to a clean black wells plate, and fluorescence was read with an excitation of 530 nm and emission of 590 nm.

#### Fluorescent staining

For calcification stainings, constructs were incubated overnight with NM containing 0.2 µmol/L of the near-infrared fluorescence bisphosphonate tracer OsteoSense 680 EX (IVISense Osteo 680 Fluorescent Prove, Revvity, Llantrisant, Ynysmaerdy, UK) in the dark. The following day, scaffolds were either washed twice in NM and imaged in NM within a glass-bottom well plate at 37 °C, 5% CO_2_ for live imaging, or taken from the inserts, washed in sterile PBS, and fixed in 3.7% formaldehyde in PBS (Sigma) for 15 min, washed twice in PBS, cut in four pieces, and stored in PBS at 4 °C for later continued staining.

For collagen staining, scaffolds were incubated in 1 µmol/L of the in-house developed collagen probe CNA35 for 1 h at 37 °C [37].

For F-actin staining, scaffolds were permeabilized using 0.5% Triton X-100 (Merck), washed with PBS, and incubated in 0.05 nmol/L phalloidin ATTO 550 (Sigma).

After calcification and collagen or F-actin staining, scaffolds were washed with PBS and incubated with 1 µg/ml 4′,6-Diamidino-2-phenylindole (DAPI, Sigma-aldrich) for 10 min. After DAPI staining, scaffolds were washed in PBS and placed in Mowiol between two coverslips, enabling imaging of both top and bottom sides of the scaffold.

Using a confocal microscope (Leica TCS SP8x, Leica, Amsterdam, Netherlands), z-stacks of at least ten slices with 5 µm spacing at three random spots of both the top and bottom sides of each construct were made, using a × 20 objective and a resolution of 1024 × 1024 µm. Z-stacks were formed to go into the scaffold as far as possible until the visual resolution was lost by interference of the scaffold fibers. Unless indicated otherwise, images shown are maximum intensity projections of five slides in z-direction from the top of the scaffold.

To evaluate FIB activation, coverslips with either FIB grown cells or NM grown cells were permeabilized using 0.5% Triton X-100 for 10 min and blocked using a solution of 2% bovine serum albumin, 5% goat serum. They were then incubated overnight in a primary antibody solution of PBS with 0.05% Tween and α-smooth muscle cell actin (α-SMA, 1:600, Mouse IgG2a, A2547, Sigma-Aldrich), vimentin (1:300, Mouse IgM, Ab20346, Abcam, Cambridge, UK), and calponin (1:600, Rabbit IgG, Ab46794, Abcam) primary antibodies. Samples were washed, incubated in secondary antibodies Alexa 555 (goat anti-mouse IgG2a), Alexa 647 (goat anti-mouse IgM), and Alexa 488 (goat anti-rabbit IggG) antibodies, incubated with DAPI for 10 min, and embedded in mowiol. Samples were imaged using a 2D fluorescence microscope (Leica, DMi8) at three locations per coverslip using a × 20 objective.

#### Calcium assay

Constructs were taken from the inserts at day 21, washed in PBS, snap frozen in liquid nitrogen, and stored at − 80 °C until processing for calcium (1/2 scaffold) or DNA and Hyp assay (1/2 scaffold). For the calcium assay, first, the wet weight was noted, after which the scaffold was lyophilized overnight, and the dry weight was measured. Then, the scaffold was wetted in 50 µl trichloroacetic acid, snap frozen, and placed in a Nalgene cryogenic vial (Sigma) containing three RNA-free metal beads. Constructs were disrupted using a dismembrator (Sartorius), for 30 s at 3000 rpm 3 times. Following this, the beads were removed, and construct remnants were incubated for 48 h in 5 wt% trichloroacetic acid (TCA, T6399, Sigma-Aldrich). Then, the calcium assay was performed according to the manufacturer’s instructions (Stanbio, 0150–250, Block Scientific). Briefly, calcium was quantified compared to a calcium chloride standard curve. Samples and standards were incubated with a cresolphthalein complexone reaction mixture, and absorbance values were read at an absorbance of 550 nm.

#### Cell and collagen amount quantification

Amount of cells within samples was quantified using DNA assay. For the DNA assay, ½ constructs were snap-frozen, wet weight was measured, and scaffolds were disrupted as described above. Cell pellets were lyophilized overnight, and dry weight was measured. Next, samples were digested in a digestion buffer consisting of 125 µg/ml papain (P5306, Sigma) in a 100 mM phosphate buffer (pH 6.5, Sigma) with 5 mM L-Cystein (Sigma) and 5 mM ethylenediaminetetraacetic acid (EDTA, Sigma) in Milli-Q at 60 °C overnight. DNA was then quantified using 10 µl of the digested supernatant using the Qubit dsDNA broad-range assay kit (Q32853, ThermoFisher), according to the manufacturer’s protocol. Hyp activity, as a derivative of total collagen amount, was quantified compared to a hydroxyproline standard curve (H5534, Sigma) using a Chloramin-T based assay (C9887, Sigma) according to the manufacturer’s protocol.

#### Real-time qPCR

pVICs cultured for FIB effectiveness analysis were lysed using RLT lysis buffer and with a sterile cell scraper taken from the plate, transferred to sterile Eppendorf’s, and stored at − 80 °C until RNA isolation using the Qiagen RNeasy kit (75,162, Qiagen, Venlo, Netherlands) according to the manufacturer’s instructions. Next, the quantity and purity of RNA were determined with a spectrophotometer (Nanodrop, ND-100, Isogen Life Science, Ijsselstein, Netherlands). cDNA synthesis was performed in a thermal cycler (C1000 Touch, Bio-rad) using a reaction mixture of 100 ng RNA in 20 µl reaction volume containing dNTPs (10,083,252, Fisher), random primers (C1181, Promega), 5 × first-strand buffer, and Moloney Murine Leukemia Virus (M-MLV, 10,338,842, Fisher). The heating cycles consisted of 65 °C (5 min); cooling on ice (2 min), during which the 5 × first-strand buffer and M-MLV were added; 37 °C (2 min); 25 °C (10 min); 37 °C (50 min); 70 °C (15 min); and cooling down to 12 °C.

To assess the efficiency of cDNA synthesis and the amount of genomic contamination in the cDNA, a conventional glyceraldehyde-3-phosphate dehydrogenase (GAPDH) check was performed and checked with gel electrophoresis.

Real-time qPCR (CFX384 Touch Real-time PCR Detection System, Bio-rad) was performed using SYBR Green Supermix (Bio-rad) and ddH2O for the α-smooth muscle cell actin (VIC activation; FW CCAGAGCAATCAGGGACC, RV TTGTCCCATTCCCACCATCA) and vimentin (general VICs; FW AGCAGTATGAGAGCGTGGCC, RV CTTCCATTTCCCGCATCTGG). Samples were diluted 30 × and run in duplicates with each gene in a thermal protocol of 95 °C (3 min), 40 cycle repeats of 95 °C (20 s), 60 °C (20 s), and 75 °C (30 s), followed by 95 °C (1 min), 60 °C (1 min), and finalized with a melting curve. The data were quantified using the delta-delta Ct method (2^−ΔΔCt^). Samples were normalized to the housekeeping genes GADPH (FW CCCAGAAGACTGTGGATGG, RV ACCTGGTCCTCAGTGTAGCC) and SDHA (FW CTACAAGGGGCAGGTTCTGA, RV AAGACAACGAGGTCCAGGAG) and further normalized to the NM control.

### Scaffold characterization

#### Scanning electron microscopy

Scaffold microarchitecture and fiber diameter were visualized using a scanning electron microscope (SEM; Quanta 600 F, GEI, Eindhoven) at high vacuum, 0.5 kV, spot size 3. On three random positions, images were taken from both the top and bottom of each scaffold at around × 1000, × 3000, and × 7000 magnification. At × 3000 magnification, 25 fibers were measured to evaluate the fiber thickness for each scaffold using ImageJ.

#### Atomic force microscopy

An atomic force microscope (JPK Nanowizard IV, Bruker, USA) was used to measure the elastic properties of the scaffolds at the single-fiber level. The measurements were performed using the quantitative imaging (QI) mode, where a force-distance (indentation) curve is acquired at each point of the scanned area. An ACLA probe (AppNano, USA) with a nominal spring constant of 58 N/m and a nominal tip radius of 10 nm was used and calibrated using the thermal noise method. Three random 50 µm × 50 µm areas of each sample were scanned by applying a set point force of 500 nN, with a pixel time of 15.0 ms and a Z length of 3.0 µm. The values of the reduced modulus E* were calculated by fitting the measured force-distance curves to the Hertz-Sneddon model, considering a paraboloid tip with a nominal tip radius of 10 nm (Eq. 2):

2 $$F={k}_{\text{c},\text{z}}{d}_{\text{c},\text{z}}=\frac{4}{3}{E}^{*}{R}_{\text{tip}}^{1/2}{\delta }^\frac{2}{3}$$

where *d*_c,z_ is the deflection of the cantilever, *k*_c,z_ the bending stiffness of the cantilever, *R*_tip_ the AFM tip radius, *δ* the indentation depth, and E* the reduced modulus of the material (Eq. 3):

3 $${E}^{*}=\frac{E}{1-{\nu }^{2}}$$

with *ν* the Poisson ratio of the material.

The reduced modulus of the single fibers was estimated as the mean value on the top central region of the visible fibers. Only surface and straight fibers were considered.

#### Differential scanning calorimetry (DSC)

Scaffold crystallinity was measured on a DSC Q2000 (TA instruments, USA). Dry scaffolds were weighed and hermetically sealed in aluminum pans (Tzero). Samples were heated to 180 °C at 40 °C/min, then cooled to − 70 °C and subsequently underwent two heating cycles from − 70 to 180 °C at 10 °C/min. The melting peak (maximum) and enthalpy (peak area) were evaluated using Universal Analysis software (V4.5A, TA Instruments).

#### Gel permeation chromatography (GPC)

Molecular weight measurements were performed on *n* = 1 sample per material per experimental repeat using GPC at a concentration of 1 mg/ml in dimethylformamide (DMF). Samples were then filtered using a 0.2 µm filter, and molecular weight was determined relative to poly(ethylene glycol) (PEG) standards, with a PL-GPC 50 Plus instrument (Varian/Polymer Laboratories, Palo Alto, CA, USA) with a Shodex GPC KD-804 column at 50 °C. *M*_n_ was defined as the number-averaged molecular weight, where *M*_w_ was defined as the weight-averaged molecular weight.

### Data analysis and statistics

For image quantification, maximum intensity projections of Osteosense, CNA, and DAPI channels, the five superficial layers of the scaffolds showing good DAPI signal were exported to Cell Profiler image analysis tool (Cellprofiler 4.2.5, Broad Institute, Boston, MA, USA) to keep quantification equal between imaging sets [38]. Within a pipeline, channels were assigned to calcification (Osteosense channel), collagen (CNA channel), or cell (DAPI) images. Images were then processed using a Gaussian (cell only) and/or median (all) filter. For DNA and calcification images, particle features were enhanced. Then, images were subjected to an experiment-depending threshold, after which calcification and collagen area were measured (in pixels), and the amount of cell objects was counted. Laser intensities changed between experiments but not within experiments. For this reason, image quantification was always normalized to the normal medium group. Experiment 2 pVIC1 was excluded from image quantification due to technical differences in imaging methods between this and other experiments.

Graphs were plotted and statistically analyzed with GraphPad Prism (version 10.1.2 (324), Boston, MA, USA). Data is shown as mean with standard deviation (SD). To evaluate differences, for samples showing a Gaussian distribution, one-way ANOVA with Bonferroni correction for multiple comparisons was used. For samples without Gaussian distribution, a Kruskal–Wallis test with Dunn’s multiple comparisons test was used. *P*-values < 0.05 were considered significantly different, and *, **, ***, **** denote *P*-values of < 0.05, < 0.01, < 0.001, and < 0.0001, respectively.

## Results

### Quiescent cells seeded onto fibrous polymer scaffolds integrate into the scaffold and form constructs in which calcification can occur after 21 days of culture

**Fig. 2:**
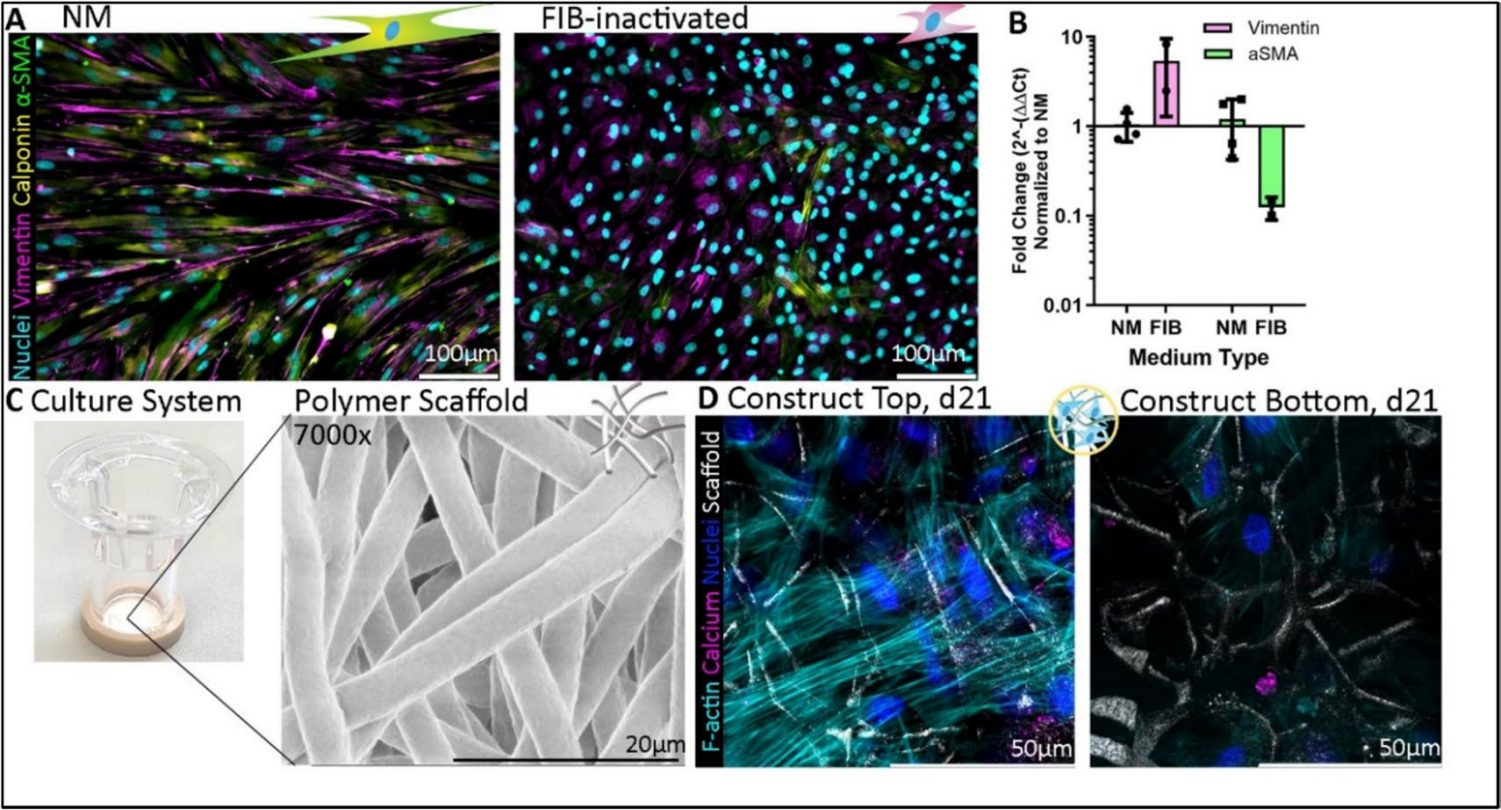
Fibrous material scaffolds allow for cell integration throughout the scaffold to form constructs that can be cultured for up to 21 days and show calcification potential. **A** Cells cultured for 7 days in FIB medium (right) showed lower expression of α-SMA (green) and calponin (yellow), as well as a more round-shaped morphology, compared to cells cultured in NM (left). **B** Expression of vimentin increased and α-SMA decreased after 7 days of culture in FIB medium (*n* = 2), compared to NM (*n* = 4). **C** Transwell system used for suspension scaffold culture and × 7000 zoom of scanning electron microscopy images of PCL scaffold as used within the model. **D** Cell integration into the scaffold after 21 days of culture (PCL Example from Experiment 3) shows calcification (pink) and F-actin positive (cyan) cells on the top (left), as well as within the bottom (right) of the scaffold, signifying cell integration throughout the scaffold. Further, a close relation between cells and scaffold fibers (white shadows), particularly at the bottom of the scaffold. NM, normal medium; FIB, fibroblast inactivation medium; α-SMA, α-smooth muscle cell actin; F-actin, filamentous actin

Similar to previous studies [32], pVICs cultured on 2D polystyrene could be turned into quiescent cells with 7 days of fibroblast inactivation medium (FIB) culture, as evidenced by a loss of α-SMA and calponin signal (Fig. 2A, Supplementary Fig. 1) and their more rounded morphology compared to pVICs cultured in NM. pVICs further showed an increase in vimentin expression and a decrease in α-SMA expression (Fig. 2B).

Fibrous scaffolds with a random fiber direction (Fig. 2C) were suspended in well plates using an in-house developed Transwell system. After seeding quiescent pVICs onto the fibrous scaffolds, cells migrated throughout the fibrous scaffold, as shown by the presence of cells within TE material constructs at the bottom of the constructs after 21 days of culture (Fig. 2D, Supplementary Fig. 2).

At the top of the constructs, cells were found spread between the fibers (Fig. 2D, left) in addition to forming a monolayer on top of the scaffold fibers, while at the bottom of the construct, cells were mainly found spread between the scaffold fibers (Fig. 2D, right). Calcification (Fig. 2D) in all groups of live-traced polymer constructs was absent after 3 days of culture, started to appear after 10 days, and increased during the 21 days of culture (Supplementary Fig. 3).

### Application I: HVTE material constructs show less calcification compared to pericardial patch control tissue

**Fig. 3:**
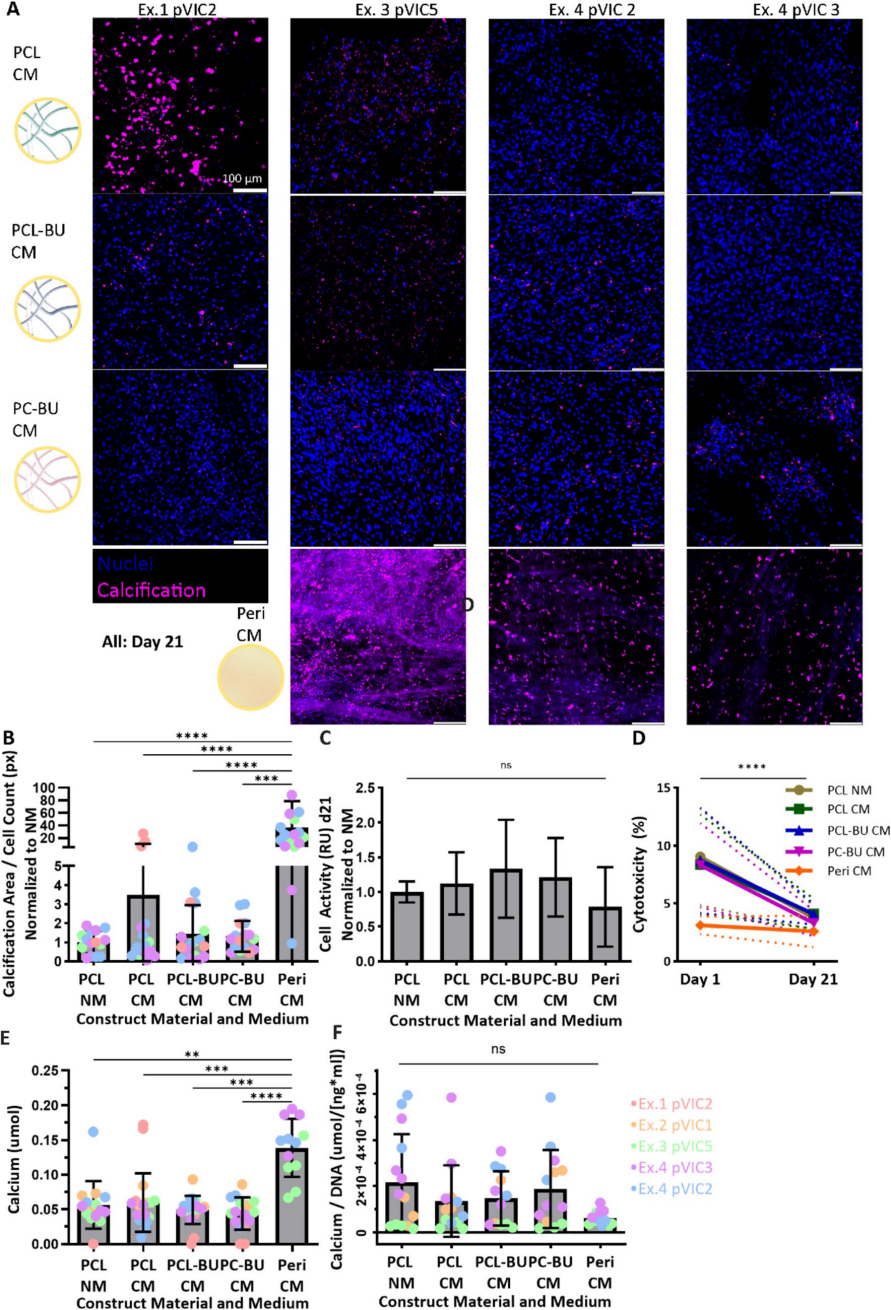
Application I: HVTE material constructs show significantly less calcification compared to pericardial patch constructs after 21 days of culture. **A** Calcification imaging using a hydroxyapatite-tracing calcium probe (pink) showed an increase in calcification within the pericardial patch control group (bottom row), compared to the three HVTE materials (top 3 rows). HVTE materials showed varying results between experiments, with an increase in calcification in PCL within experiments 1 and 2 (top row, first two columns), and an even distribution of calcification in experiments 3 and 4 (right three columns) between the HVTE materials. **B** Quantification of imaging for calcification per cell count confirmed the results shown in **A**. **C** Presto blue analysis showed no significant difference in cell activity between groups after 21 days of culture. **D** LDH assay results showed 8–9% cell death directly after cell-seeding onto HVTE scaffolds and 3% after seeding onto pericardial patch tissue, with a significant decrease in cytotoxicity within all groups to 2.5–4% after 21 days of culture. Represented as mean (line) + standard deviation (dotted line) **E** Calcium quantification using a cresolphthalein complexone based calcium assay of total calcium further showed a significant increase in calcification of pericardial tissue compared to HVTE constructs. **F** Calcium assay quantification normalized to DNA showed no significant difference in calcium/DNA between groups. PCL, polycaprolactone; PCL-BU, bisurea chain extended PCL; PC-BU, bisurea chain extended polycarbonate; Peri, pericardial patch tissue; CM, β-glycerophosphate-based calcification medium; NM, normal medium; Ex, experiment; pVIC, porcine valvular interstitial cells; NS, not significant; **p < 0.01; ***p < 0.001; ****p < 0.0001

The model was applied to study the difference in calcification potential between HVTE materials and a clinically relevant control material (bovine pericardial tissue). Control bovine pericardial tissue (Peri) showed a significant increase in calcification compared to HVTE constructs in several repeat experiments. This was demonstrated using imaging with a hydroxyapatite-tracing calcium probe (Fig. 3A, bottom row compared to top three rows) and quantified by image calcified area/cell count (Fig. 3B). Calcium quantification using a cresolphthalein complexone assay also showed a significant increase in calcium for pericardial patch tissue (Fig. 3E), but not when corrected for the amount of DNA (Fig. 3F), due to the vast amount of DNA remnants in the pericardial patch tissue (Supplementary Fig. 4 F, see next “Results” section).

When comparing HVTE materials, differences in calcification between materials were confounded by the differences between experiments. There was a trend towards enhanced calcification in the PCL and PCL-BU scaffolds in the first experiment, but more calcification in the PC-BU scaffolds in the last repeat (Fig. 3A, B, E, F, Supplementary Fig. 4D). This was not caused by the pVIC donor, as experiments 1 and 4 used the same donor (pVIC 2) with opposing results.

Presto blue cell activity showed no significant differences between materials at day 21 of culture (Fig. 3C), indicating the presence of equal amounts of viable/active cells on all materials at day 21. Cell death, as measured by the LDH cytotoxicity assay, was comparable between different materials (Fig. 3D), with an initial 8–9% cell death 1 day after seeding the HVTE constructs, and 3.1% cell death after seeding on pericardial tissue. There was no significant difference between HVTE and pericardial tissue constructs. Cytotoxicity significantly decreased in all materials from day 1 until day 21 of culture, with 2.5–4% cytotoxicity remaining, suggesting that the initial effect is mainly caused by the cell-seeding process rather than the cytotoxicity of the materials.

Briefly, the model allowed for the induction and assessment of variations in calcification between experiments and materials, with less calcification found in HVTE constructs compared to pericardial control tissue.

### Calcification within HVTE constructs is correlated to cellularity, whereas calcification in pericardial patch tissue is not, and HVTE constructs show an equal potential to form collagen

**Fig. 4:**
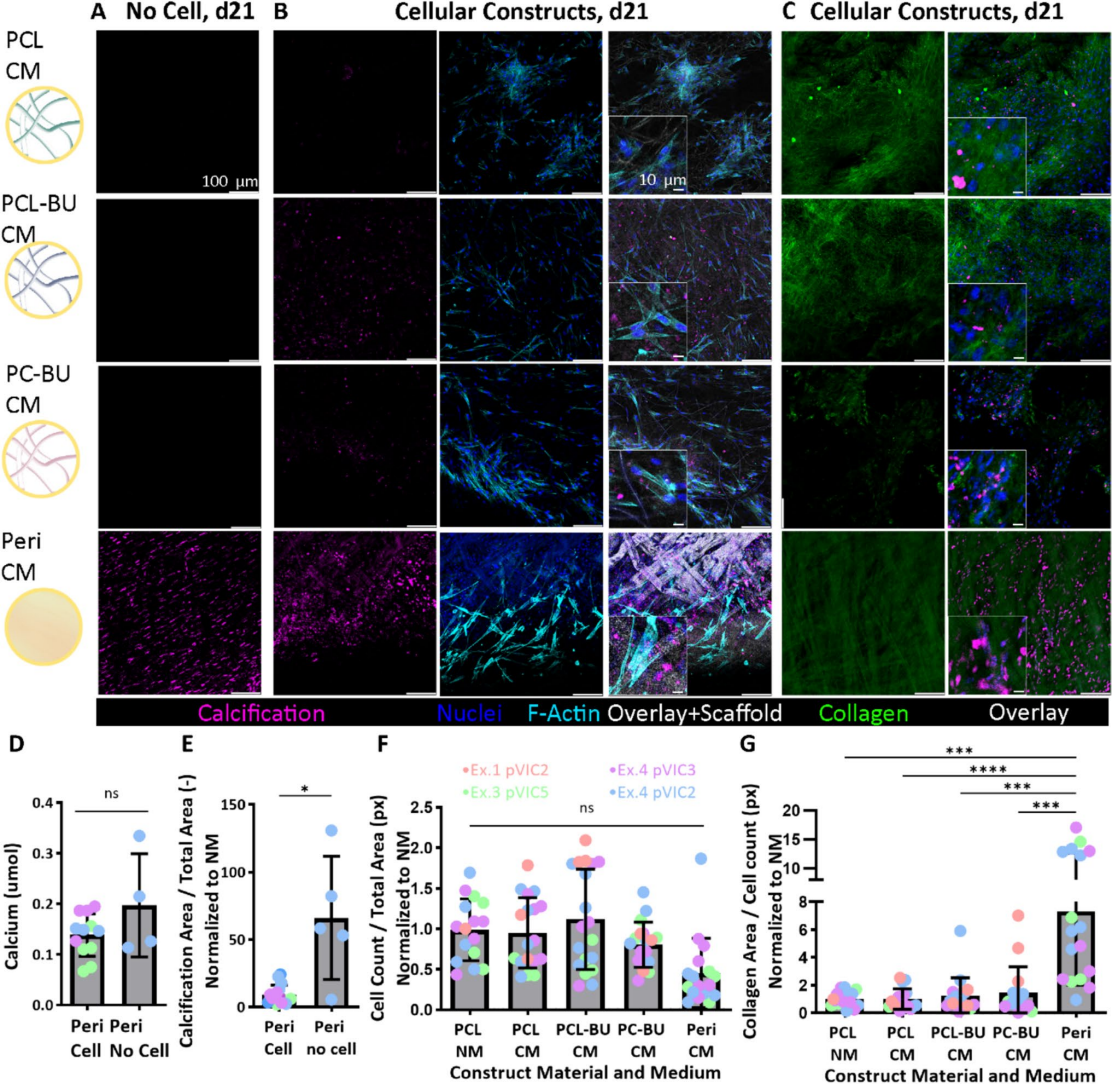
Correlation of cellularity, collagen formation, and calcification shows cell-dependent calcification in HVTE constructs but not in pericardial tissue, and equal cell-dependent collagen formation within HVTE constructs. **A** Bare, acellular scaffolds cultured for 21 days in CM showed no calcification (pink) in HVTE materials (top three), but significant calcification in pericardial tissue (bottom). Representative maximum intensity projections. **B** Calcification in HVTE constructs (top three) was colocalized with F-actin (cyan) positive cell locations, whereas in pericardial tissue, calcification was found in F-actin positive and negative areas (bottom). Representative single z-slice images*.* **C** Collagen staining (green) in combination with nuclei (dark blue) and calcification (pink) showed more aligned, dense collagen in pericardial tissue compared to HVTE constructs, and calcification particles seemed not to be integrated into the collagen layer. Representative single z-slice images. **D** Calcium assay quantification showed similar calcium levels between pericardial tissue, independent of cell seeding, and **E** image calcium quantification showed an increase in calcium particles within bare, unseeded pericardial tissue compared to seeded tissue. **F** Cell count imaging quantification showed similar cell distribution between all materials. **G** Collagen area per cell count imaging quantification showed a significant increase in collagen within pericardial tissue compared to HVTE constructs, without difference between HVTE constructs. PCL, polycaprolactone; PCL-BU, bisurea chain extended PCL; PC-BU, bisurea chain extended polycarbonate; Peri, pericardial patch tissue; d21, day 21 of cell culture; CM, β-glycerophosphate-based calcification medium; NM, normal medium; Ex, experiment; pVIC, porcine valvular interstitial cells; NS, not significant; *p < 0.05; ***p < 0.001; ****p < 0.0001

To look at the correlation of calcification with cellularity, acellular scaffolds were cultured in CM for 3 weeks. HVTE materials showed no calcification when cultured in the absence of cells (Fig. 4A, top three). Furthermore, F-actin staining showed F-actin positive cells at the top as well as the bottom of cell-cultured material scaffolds after 21 days (Supplementary Fig. 2) and co-localization of calcification and cells in material scaffolds (Fig. 4B). In contrast, pericardial calcification was also found in non-cell-seeded cultured pericardium (Fig. 4A, bottom) in similar amounts to cell-seeded pericardium (Fig. 4D, E). Unlike HVTE material constructs, cell migration to the bottom of the pericardial tissue was limited due to the tissue’s density (hence, no F-actin positive cells in the bottom as seen in Supplementary Fig. 2), but calcification was still found at the bottom. Non-cell-seeded and non-cultured pericardium did not show calcification (Supplementary Fig. 4A), indicating that pericardium calcification depended on exposure to the β-glycerophosphate-rich medium but was independent of the presence of seeded cells.

Further looking at morphological characteristics of cells cultured within the HVTE constructs, F-actin expression showed a spindle-shaped cell morphology (Fig. 4B). F-actin expression did not follow a specific direction or pattern; instead, cells spread randomly throughout the scaffold and between scaffold fibers (Figs. 4B and 2D). F-actin-positive cells were found on both the top and bottom of the HVTE constructs. Interestingly, pericardial scaffolds showed an F-actin positive layer on the top of the material (possibly the seeded cells), which was lost within the first layers of tissue, where the nuclear DAPI signal was still present (possibly DNA remnants). Pericardial tissue did not show F-actin expression on the bottom of the scaffold, likely due to the absence of live cell migration throughout the dense pericardial tissue (Supplementary Fig. 2). Cell count image quantification showed no significant difference between materials (Fig. 4F), but a DNA assay showed significantly more DNA within the pericardial tissue (Supplementary Fig. 4F), with DNA present in unseeded pericardial tissue as well. This further signifies the presence of old cell remnants/DNA within the pericardial tissue. Calcification within the pericardial tissue aligned well with the presence of nucleic parts but not with phalloidin-positive cells (Fig. 4A, bottom), indicating cell-seeding independent nucleation of calcium on DNA remnants within the pericardial tissue.

Collagen synthesis was similar for all cell-seeded HVTE materials and experiments, with cells producing abundant layers of randomly distributed (non-aligned) collagen (Fig. 4B, G). Calcification was not integrated within the collagen (Fig. 4C) but formed as loose particles separate from the collagen fibers. As expected from its mechanical function, pericardial tissue showed more organized collagen. The Hyp assay was not sensitive enough to detect collagen levels within HVTE; only within pericardial tissue, Hyp contents of 1.6 µg/[ml*mg sample weight] (+/− 1.5 µg/[ml*mg]*)* were found (Supplementary Fig. 4G).

### Scaffold characterization shows that HVTE materials are composed of similar fiber diameters but with different stiffness and enthalpy, with little differences between experiments

Scaffold materials were further characterized to determine if differences in material properties could explain the observed trends in calcification variation between experiments. Specifically, more calcification was noted in the PCL and PCL-BU scaffolds in the first two experiments, equal amounts in the third, and more calcification in the PC-BU scaffolds in the last experiment (Fig. 3A, B, E, F).

**Fig. 5:**
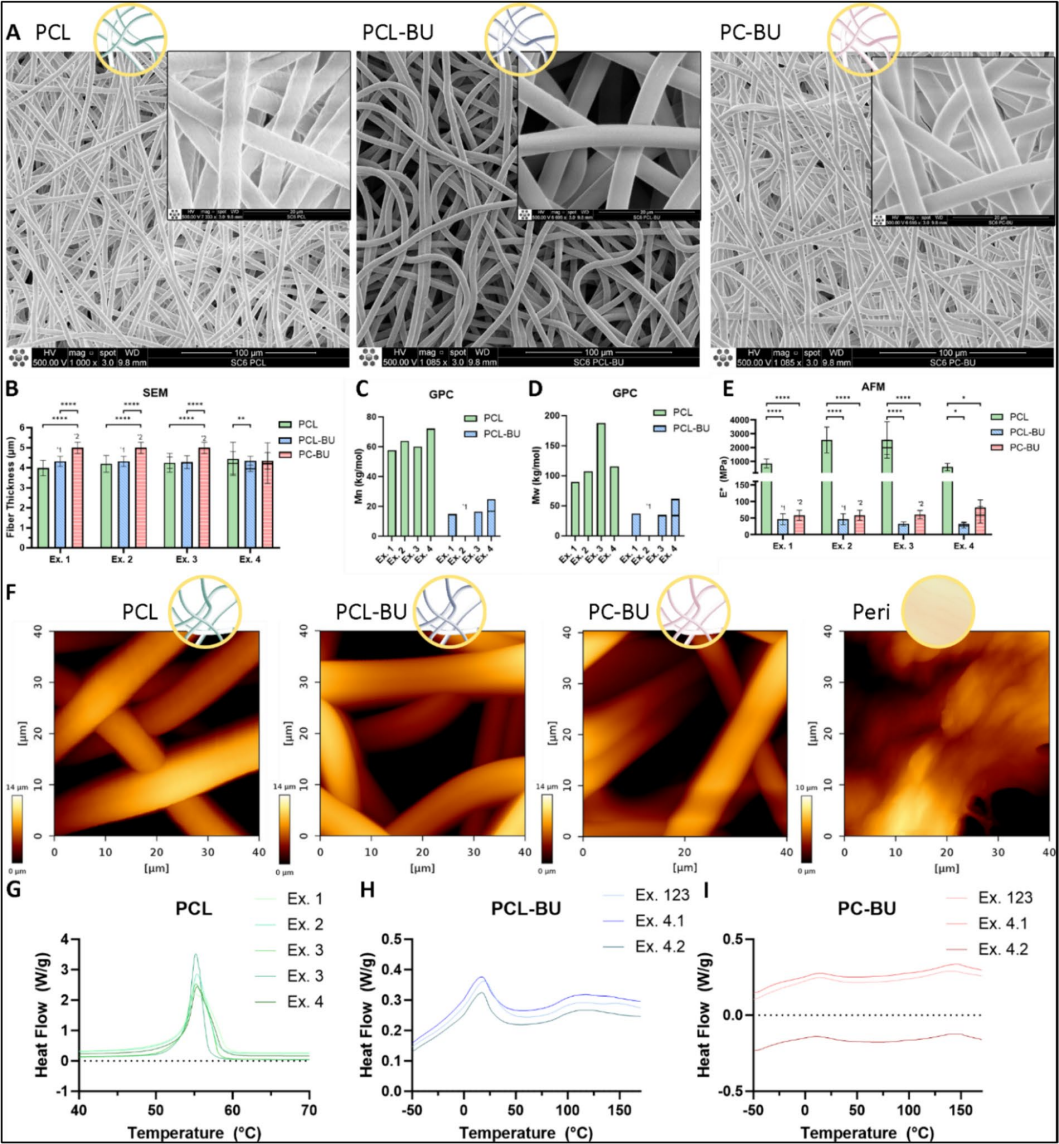
Scaffold properties do not explain inter-experimental differences in material calcification. **A** Scaffolds were evaluated using scanning electron microscopy (SEM) to show fiber diameter and morphology. **B** Fiber diameter quantification showed a similar fiber distribution, with a significantly higher fiber diameter of PC-BU in the first three experiments. **C** Molecular number was equally distributed within PCL and PCL-BU materials throughout experiments (*n* = 1). **D** Molecular weight of PCL was slightly higher within experiment 3 and otherwise equally distributed throughout experiments (*n* = 1). **E** Fiber-reduced modulus evaluation showed a higher reduced modulus of PCL fibers compared to PCL-BU and PC-BU, with a slight increase within experiments 2 and 3. **F** AFM evaluated fiber morphology showed similar fiber characteristics compared to SEM evaluation. **G** DSC data showed a single melting peak in PCL and a double peak in **H** PCL-BU as well as **I** PC-BU that remained unchanged between experiments. PCL, polycaprolactone; PCL-BU, bisurea chain extended PCL; PC-BU, bisurea chain extended polycarbonate; SEM, scanning electron microscopy; GPC, gel permeation chromatography; Mn, molecular number; Mw, molecular weight; AFM, atomic force microscopy; DSC, differential scanning calorimetry; LDH, lactate dehydrogenase; IF, immunofluorescence; Ex., experiment; NS, not significant; *p < 0.05; **p < 0.01; ****p < 0.0001

Before the experiments, electrospinning parameters were optimized to achieve fibers approximately 4 µm in thickness for all scaffolds (Fig. 5A, B, Table 2, Supplementary Fig. 5). Notably, in the first three experiments, the PC-BU scaffolds had significantly thicker fiber diameters (4.99 µm, SD 0.28 µm; Fig. 5B, Supplementary Fig. 5), though this was only a 1 µm difference compared to the other materials (3.99–4.25 µm for PCL and 4.32–4.25 µm for PCL-BU). Since fiber diameter is linearly related to pore size [39], it could be assumed that pore size was also increased in PC-BU within the first three experiments. This did, however, not influence cell integration into the bottom of the scaffold (Supplementary Fig. 2). In Experiment 4, all fiber diameters were similar.

**Table 2:** Average scaffold characteristics

		Fiber thickness		Reduced modulus		Molecular weight scaffold			Melting peak		Melting enthalpy	
	Material	*T* (µm)	SD *T* (µm)	E* (MPa)	SD E* (MPa)	Mn (kg/mol)	Mw (kg/mol)	*Ð*	Tm1 (°C)	Tm2 (°C)	Δ*H*m1 (J/g)	Δ*H*m2 (J/g)
Experiment average	**PCL**	4.19	0.47	1566.2	609.2	63.4	125.3	2	55.2	NA	64.2	NA
	**PCL-BU**	4.27	0.25	38.7	11.6	18.3	42.0	2.3	19.0	111.5	13.1	12.0
	**PC-BU**	4.82	0.39	62.0	16.2	Not measurable			13.2	144.9	3.6	10.4

Weight-averaged and number-averaged molecular weights (*M*_w_ and *M*_n_) could only be evaluated for PCL and PCL-BU due to the inability of PC-BU to dissolve in dimethylformamide or tetrahydrofuran. *M*_w_ and *M*_n_ of PCL were higher than those of PC-BU (Fig. 5C, D, Table 2). The *M*_n_ of both materials was similar across all experiments, but the *M*_w_ was slightly higher in PCL in Experiment 3 compared to the other experiments (Fig. 5C, D, Supplementary Table 2).

AFM showed a significantly higher reduced modulus of PCL fibers compared to PCL-BU and PC-BU fibers (Fig. 5E, F, Table 2), without significant differences in materials between experiments (Fig. 5E, Supplementary Table 2).

DSC plots showed two melting transitions for PC-BU and PCL-BU materials, with one originating from the PC or PCL polymers and the other peak resulting from the stacking of the BU. Only one melting transition was observed for PCL material (Fig. 5G–I). The melting peaks and enthalpy of all materials did not differ between experiments, indicating that crystallinity was not significantly different between experiments.

Therefore, materials varied in terms of fiber diameter, molecular number, molecular weight, reduced modulus, and melting transitions. However, these variations did not cause differences in calcification between materials and did not vary significantly within materials between experiments.

### Application II: cyclic straining of HVTE constructs does not change the calcification potential of pVIC-loaded PCL-BU and PC-BU constructs

**Fig. 6:**
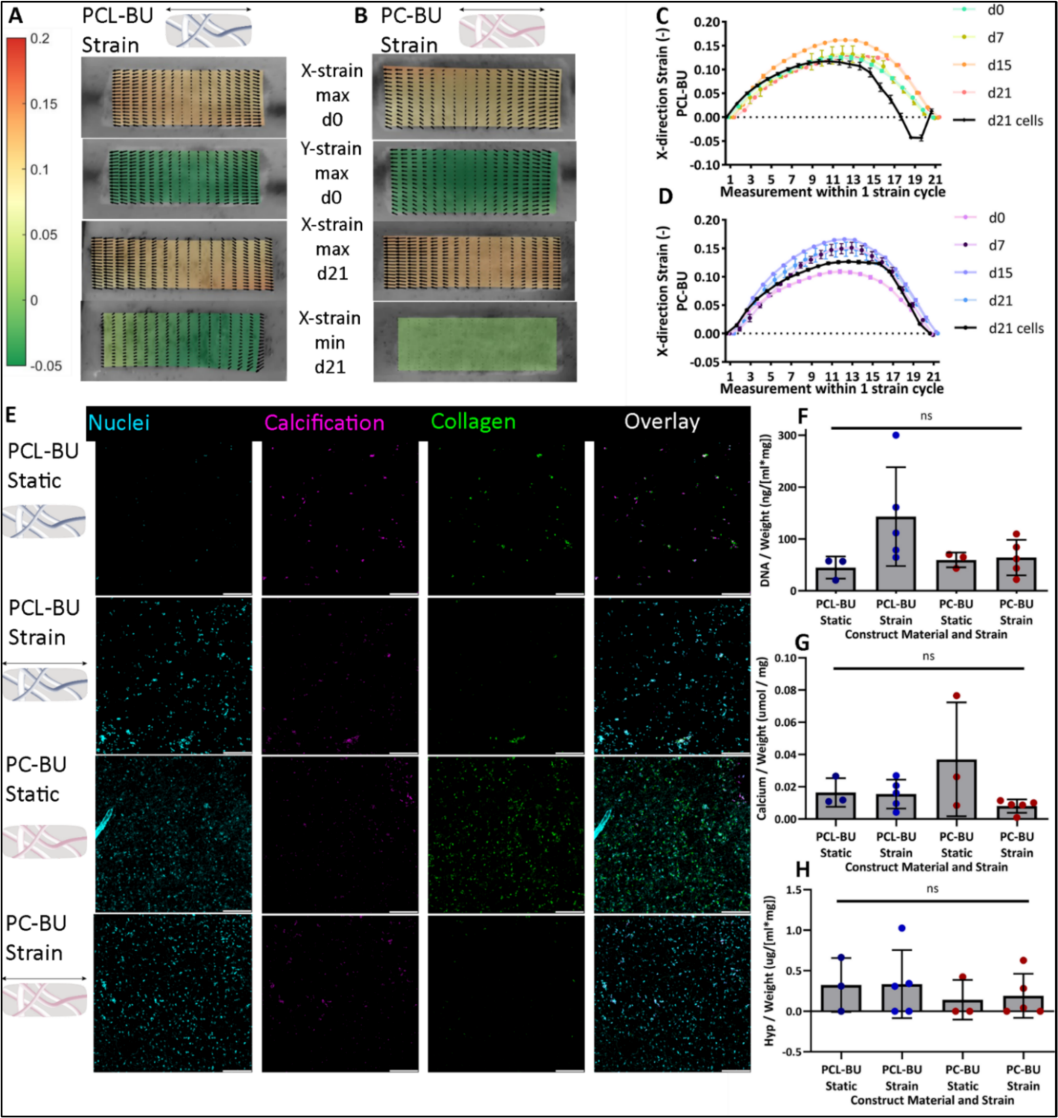
Application III: application of cyclic strain to pVIC-loaded HVTE constructs does not significantly change their calcification potential. **A**, **B** Cyclic strain analysis of PCL-BU (**A**) and PC-BU (**B**) scaffolds in the *x*-direction at day 0 (before the start of the experiment, top), in the *y*-direction at day 0, and the maximum and minimum *x*-directional strain of cell-cultured scaffolds at day 21 of the experiment show an *x*-directional strain at day 0 within both materials and material fatigue over time, as indicated by a negative minimum strain at day 21 within PCL-BU material (**A**, bottom). **C**, **D** The maximum strain applied to the constructs was 10–15% and did not change throughout the 21-day culture period. The minimum strain stayed at 0% within cell-free scaffolds. Within cell-loaded constructs, a minimum strain of − 0.5% was found within PCL-BU scaffolds at day 21. **E** Calcium imaging shows no significant differences in calcification between constructs in strained or static conditions. **F** DNA assay showed a trending but insignificant increase in DNA in stretched materials. **G** Calcium assay showed no significant difference between strained and unstrained materials. **H** Hyp assay shows no significant differences in collagen formation between strained and static conditions, with a trending lower amount of collagen in PC-BU material constructs. PCL-BU, bisurea chain extended PCL; PC-BU, bisurea chain extended polycarbonate; Hyp, hydroxyproline assay; ns, not significant

In a pilot to show possible applications of the model, we tested the influence of cyclic tensile strains of physiological magnitude on HVTE construct calcification. The applied 10% Flexcell strain resulted in a maximum in-plane strain (x-direction) of 10.53% (SD 0.33%) in PC-BU and 12.51% (SD 0.22%) in PCL-BU at day 0, which varied over time throughout the experiment, likely due to the material fatigue of the scaffolds (Fig. 6A–D). The strain in the *y*-direction was negative, nearing 0 at all time points (Fig. 6A, B), indicating minor tissue contraction during stretch in the *x*-direction. Strain did not change the calcification potential of cells within either material (Fig. 6G). Hydroxyproline (HYP) quantification showed no significant differences between static or stretched constructs, although HYP levels were slightly higher in PCL-BU scaffolds compared to PC-BU scaffolds (Fig. 6H).

In brief, applying cyclic strain to the materials did not affect calcification or tissue remodeling by pVICs alone.

## Discussion

In this study, we developed and tested a 3D in vitro model to assess calcification in HVTE scaffolds prepared from synthetic polymeric materials. These scaffolds were cultured for 3 weeks to form engineered constructs. Selected electrospun fibrous scaffolds, cultured in a suspension platform with porcine valvular interstitial cells (pVICs) and a calcification-permitting medium (CM), showed significantly less (micro-) calcification than a clinically used material, a bovine pericardial patch. This data aligns with in vivo results, as the previous systematic review showed that calcifications in HVTE valve implants mostly consisted of micro or mild nodules (in 76% of cases), and the degree of calcification was always less pronounced than in bioprosthetic valve controls [19]*.* Our model addressed the important hallmarks for a calcification model of in situ HVTE (Table 3), with the inclusion of (1) 3D polymer scaffolds, (2) a relevant cell source, (3) a calcification-permitting medium and relevant outcome parameters, (4) a clinically relevant control material, and (5) the ability to capture in vivo-like risk factors for calcification (hemodynamics). Each of these hallmarks contains certain experimental choices with limitations and advantages.

**Table 3:**
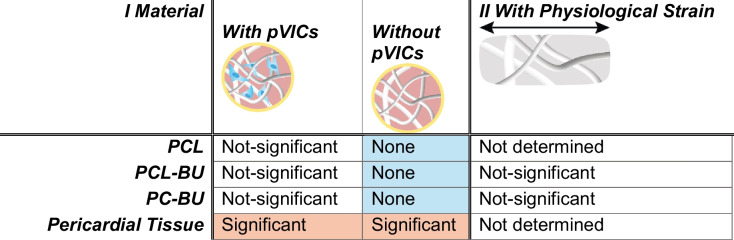
Calcification differences found using model application

All after 21 days of culture in Calcification-permitting medium

The impact of 3D polymer scaffold material on calcification was investigated by comparing various synthetic materials to pericardial patch tissue, which is commonly used in clinical valve implants. We observed a significant increase in calcification within the pericardial tissue. Previous in vivo preclinical studies using HVTE materials in sheep models demonstrated that synthetic material induced lower calcification compared to valves made from glutaraldehyde-treated porcine or bovine tissue [13, 15]. Our model shows a similar effect, suggesting it is a relevant model for studying HVTE materials and predicting their (pre-) clinical response.

In this study, we observed some inter-experiment variation in the calcification of synthetic material constructs. This variation could not be attributed to donor differences, as it persisted even when experiments were repeated using the same pVIC donor. Despite extensive testing, we could not identify a conclusive material-based cause for this variation. One possibility is that solution preparation before electrospinning influences calcification in ways not explained by modulus and fiber morphology, highlighting the importance of consistent polymer solution preparation. Another tested explanation was a slight difference in material degradation due to different storage methods between the first (RT) and subsequent experiments (− 20 °C). In vivo, differences in material degradation have been linked to variations in vascular graft calcification [40]. However, we found no significant difference in material degradation, as indicated by the similarities in crystallinity and molecular weight of the materials. Future studies should investigate whether material degradation impacts calcification in these materials. It should be noted that inter-experiment variation of calcification within materials limits the robustness of the model. However, experimental variations in calcifications have also been found in vivo in animal models for HVTE [41]. In our study, these differences did not contribute to significant differences between experiments, though these variations should be further studied.

As a relevant cell source for calcification, we opted for cultures with and without porcine valvular interstitial cells (pVICs). pVICs have been widely used to study valvular biology and calcification, showing similar potential to human VICs in terms of calcification and differentiation potential in vitro [31, 42]. Our study reaffirmed that pVICs are effective for studying valvular calcification and serve as an easy cell source for high-throughput 3D models requiring large quantities of cells. While human VICs would make these models more clinically relevant, their slower growth rate compared to porcine VICs could hinder upscaling [42]. One limitation of using (p)VICs in HVTE research is that after valve implantation, the native valves might be excised. Aortic smooth muscle cells could provide another cell source, as these will grow into the regenerating valve tissue [43]. Alternatively, induced pluripotent stem cells (iPSCs) could provide a viable cell source, overcoming the challenges of large cell quantities needed for 3D culture and offering a basis for a human-translatable model with minimal donor-to-donor variation [44, 45]. Studying donor-to-donor variation in relation to materials can be insightful, as it might influence both in vitro [46] and in vivo results [47]. However, to understand the fundamentals of (disadvantageous) material remodelling, especially when considering different material characteristics and batch differences, it might be optimal to simplify in vitro systems by minimizing donor-to-donor variation.

Various medium components have been used to study calcification, including organic phosphate (β-glycerophosphate, βGP), L-ascorbic acid (AA), and dexamethasone, which enable calcium formation in pVICs by transforming the cells to an osteoblastic phenotype [48–50]. Inorganic phosphate (NaH2PO4, PI) has been shown to allow for a more severe calcification potential [42, 51] but has been shown to be alkalic phosphatase (ALP) independent [52]. Elevated calcium levels in the medium also increase calcification through cellular osteogenic differentiation [53, 54]. These additives model disease processes by inducing osteogenic differentiation (pathological cell differentiation) or creating a hyperphosphate/calcium environment (as for example in renal failure). In the presented model, we aimed to create a platform to study external effects on calcification within HVTE materials, without creating a disease model. We therefore opted to use an increased concentration of β-glycerophosphate only. A limitation of this choice could be that it adds to the inter-experimental variation. The limited degree of calcification formation within the TE material scaffolds may impede the comparison of currently non-significant material differences. However, an increase in osteogenic cell-driven calcification could yield erroneous conclusions. Future studies should elucidate whether other external (scaffold-related) factors, such as hemodynamics presented herein, can influence calcification. For instance, the impact of inflammation on the calcification of synthetic constructs could be studied, as previous studies have established inflammation-driven calcification mechanisms in valvular disease modeling [21, 55]. Wissing et al. previously identified differences in macrophage response to different HVTE material scaffold microarchitectures [22]. Our current model could be extended to study the effect of varying macrophage responses on VIC-mediated ECM remodeling and calcification.

In this study, we used pericardial tissue as a clinically relevant control since valves made from this tissue are currently used as biological valve transplants [56]. Multiple studies using in vivo animal models for HVTE remodeling previously compared their TE materials to glutaraldehyde-fixed valvular implants [13, 57]. It should be noted that the mechanisms of calcification between HVTE materials and pericardial tissue are not to be compared. When comparing calcification results with and without pVICs, we observed no calcification in HVTE materials without cells, indicating that calcification in synthetic materials is likely an active cell-mediated response. Pericardial tissue, however, showed calcification both in the presence and absence of cells. Schoen et al. previously described glutaraldehyde-fixed tissue calcification as passive calcium phosphate precipitation on DNA remnants within devitalized tissues [58]. Our model demonstrated that calcification within pericardial tissues was formed by a similar passive response, as calcification was found even after culture in the absence of pVICs. When comparing cellular reactions to native tissue to synthetic in situ HVTE materials, future studies should integrate tissues like decellularized valves or decellularized pericardial tissue [59], or decellularized valvular tissue [60], to further characterize (potential) calcification mechanisms in the absence of passive calcium phosphate precipitation.

As a risk factor for calcification, we evaluated if strain could increase the calcification potential of cells within our HVTE materials. Physiological cyclic strain did not influence calcification within HVTE material constructs in this study. Previous studies have shown that 10%, 15%, and 20% strains increase VIC intracellular calcium in 2D compared to static conditions [61, 62]. However, Ferdous et al. observed a decrease in alkaline phosphatase expression of VICs cultured under 10% strain compared to 15% strain [62]. Using our current model, we speculate that either the strain levels of 10% were modulated by material plasticity to sub-pathological strain levels or that more osteogenic-inducing medium components are needed to induce calcium differences at these strain levels [61]. Further studies should investigate whether a macrophage phenotype switch in the presence of hemodynamic cyclic strain, as previously shown [63], can affect material differences within HVTE calcification. Pericardial tissue and PCL material were excluded from strain analysis due to their material properties, which render them incompatible. PCL scaffolds were incapable of withstanding the 10% strain imposed by the Flexcell. Furthermore, the attachment materials to the Flexcell membranes necessitate the use of adhesive glue, which dries overnight (methods). The drying process dehydrates the pericardial tissue, thereby compromising the integrity of the material. The supramolecular polymer scaffolds, namely PCL-BU and PC-BU, can be adhered without issues. The breakage strain of these materials is also significantly higher than that of PCL [64], hence their preferred application for valvular tissue engineering [16, 41]. These materials were therefore solely compared in the cyclic strain analysis.

Our model demonstrated that pVICs are necessary to form (micro-) calcifications within constructs and that, similar to valvular calcification, this process can be influenced by inflammation. We also observed that, like valvular calcification, mineral particles appeared to be isolated from collagen fibers. HVTE material construct calcification seemed distinct from bioprosthetic material calcification, but further studies should explore the mechanistic similarities or differences between HVTE calcification and CAVD.

In conclusion, we established a model to study calcification in synthetic materials. This model has shown that (1) HVTE materials at baseline have a lower propensity to calcify than those currently used in clinical valve implants (pericardial patch); (2) HVTE calcification is an active cell-mediated process, whereas pericardial patch calcification is a passive process; and (3) physiological cyclic strains do not influence the calcification of HVTE materials in the presence of pVICs alone. This model can be used to study mechanisms underlying tissue remodeling and calcification within in situ HVTE and can inform the production and testing of new materials for in situ HVTE.

## Supplementary Information

Below is the link to the electronic supplementary material.

Supplementary file1 (PDF 2964 KB)

## Data Availability

The data that support the findings of this study are not openly available due to reasons of confidentiality and are available from the corresponding author upon reasonable request.
