# Supplementary material for: In vitro model assesses the susceptibility of polymeric scaffolds for material-driven heart valve regeneration to calcification
Source: In Vitro Model. 2025 Jul 15;4(2):157–75. doi: 10.1007/s44164-025-00090-x (PMC12283539; doi:10.1007/s44164-025-00090-x)
Supplement: Supplementary file 1 — Supplementary file1 (PDF 2964 KB) [file 44164_2025_90_MOESM1_ESM.pdf]

Supplementary Figures and Tables

In vitro model assesses the susceptibility of polymeric scaffolds for material-driven heart valve regeneration to calcification

Dewy C. van der Valk, MD[1,2], Charlotte M. Hoes, MSc[1], Yunia M.H. Rasenberg, MSc[1], Paul A.A. Bartels, PhD[2,3], Livia Angeloni, PhD[1,2], Bente J. de Kort[1,2], Paul J.F.M. Janssen, PhD[3], Frank, P.T. Baaijens, PhD[1,2], Anthal I.P.M. Smits, PhD[1,2], Carlijn V.C. Bouten, PhD[1,2*]

1 Department of Biomedical Engineering, Eindhoven University of Technology, Eindhoven, The Netherlands
2 Institute for Complex Molecular Systems (ICMS), Eindhoven University of Technology, Eindhoven, The Netherlands
3 Department of Mechanical Engineering, Eindhoven University of Technology, Eindhoven, The Netherlands

* Corresponding Author. E-mail: C.V.C.Bouten@tue.nl

As submitted to: *In Vitro Models*

Legend:

Supplementary Figure 1. FIB inactivated pVICs Page 2

Supplementary Figure 2. Cell migration into the scaffold Page 3

Supplementary Figure 3. Live Calcium tracing over a 21 day culture of pVICs within HVTE material constructs Page 4

Supplementary Figure 4. Pericardial tissue culture and calcification, DNA, and Hyp assays Page 5

Supplementary Figure 5. SEM imaging of HVTE scaffolds over several experiments Page 6

Supplementary Table 1. pVIC and scaffold Sheet used per experiment. Page 7

Supplementary Table 2. Scaffold characterization per experiment Page 7

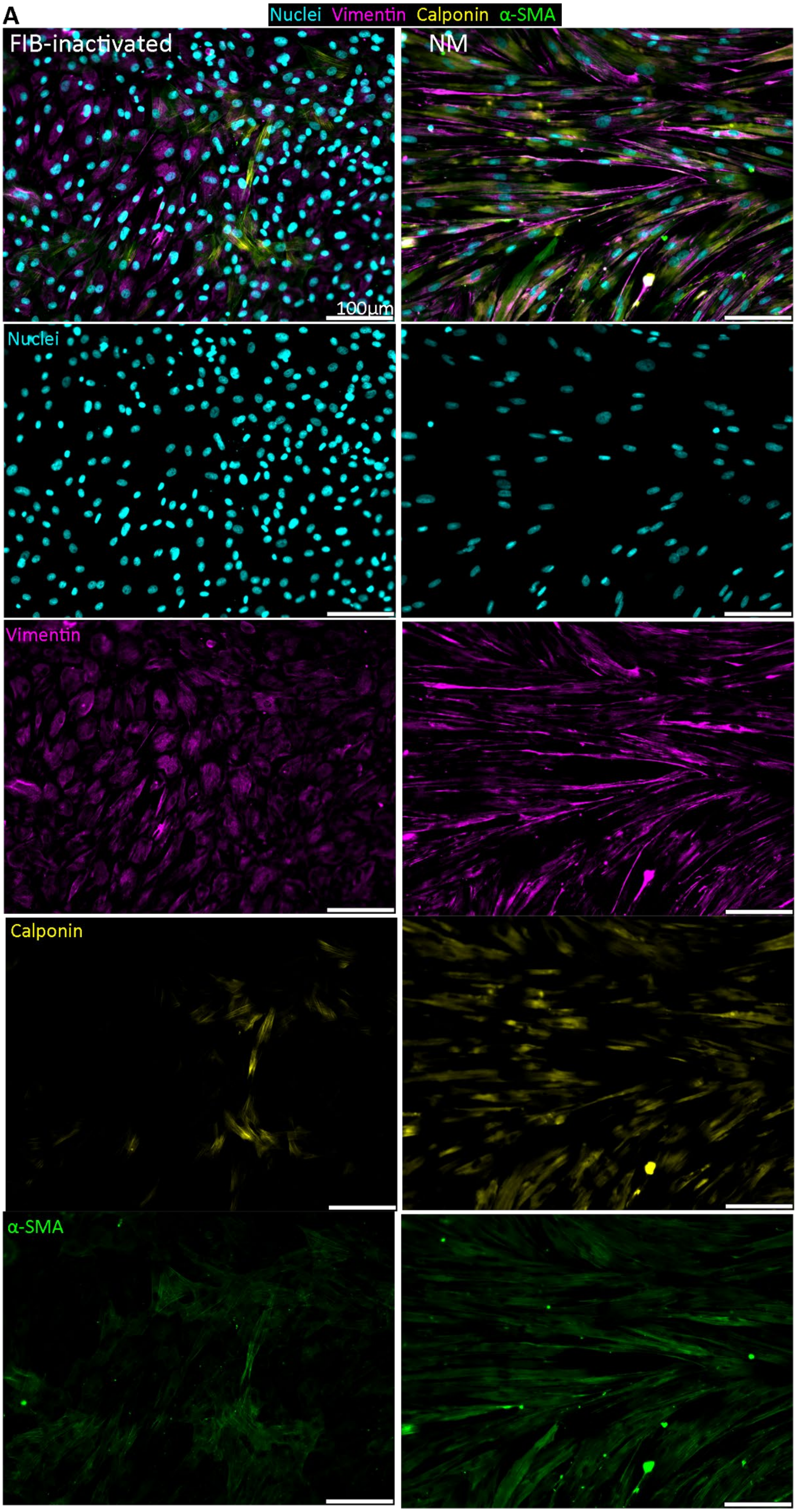

Supplementary Figure 1. FIB inactivated pVICs show a distinct rounded morphology, and a more quiescent phenotype as indicated by the lower amount of α-SMA (green) and Calponin (yellow) staining, with similar amounts of Vimentin. *NM: Normal Medium; FIB: Fibroblast Inactivation Medium; α-SMA: α-smooth muscle cell actin; F-actin: filamentous actin.*

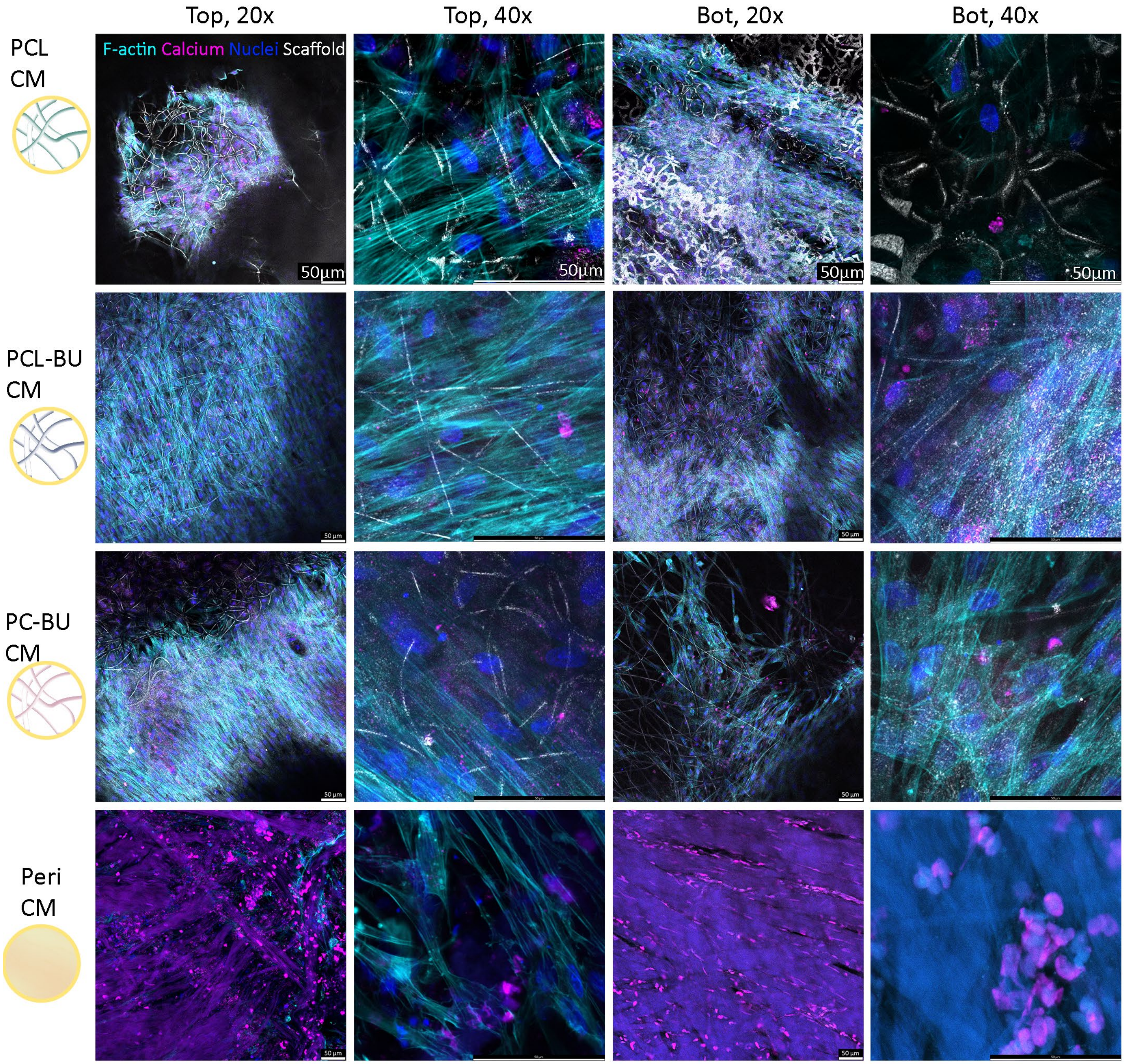

Supplementary Figure 2. Cell migration into the scaffold after 21 days of culture shows F-actin positive cells (cyan) around calcification (pink) in material scaffolds (top 3 rows) in the top of the scaffold (left two columns), as well as in the bottom of the scaffold (right two columns). The pericardial tissue (bottom row) showed calcification, but not specifically surrounding F-actin positive cells, and no F-actin positive cells in the bottom of the scaffold.

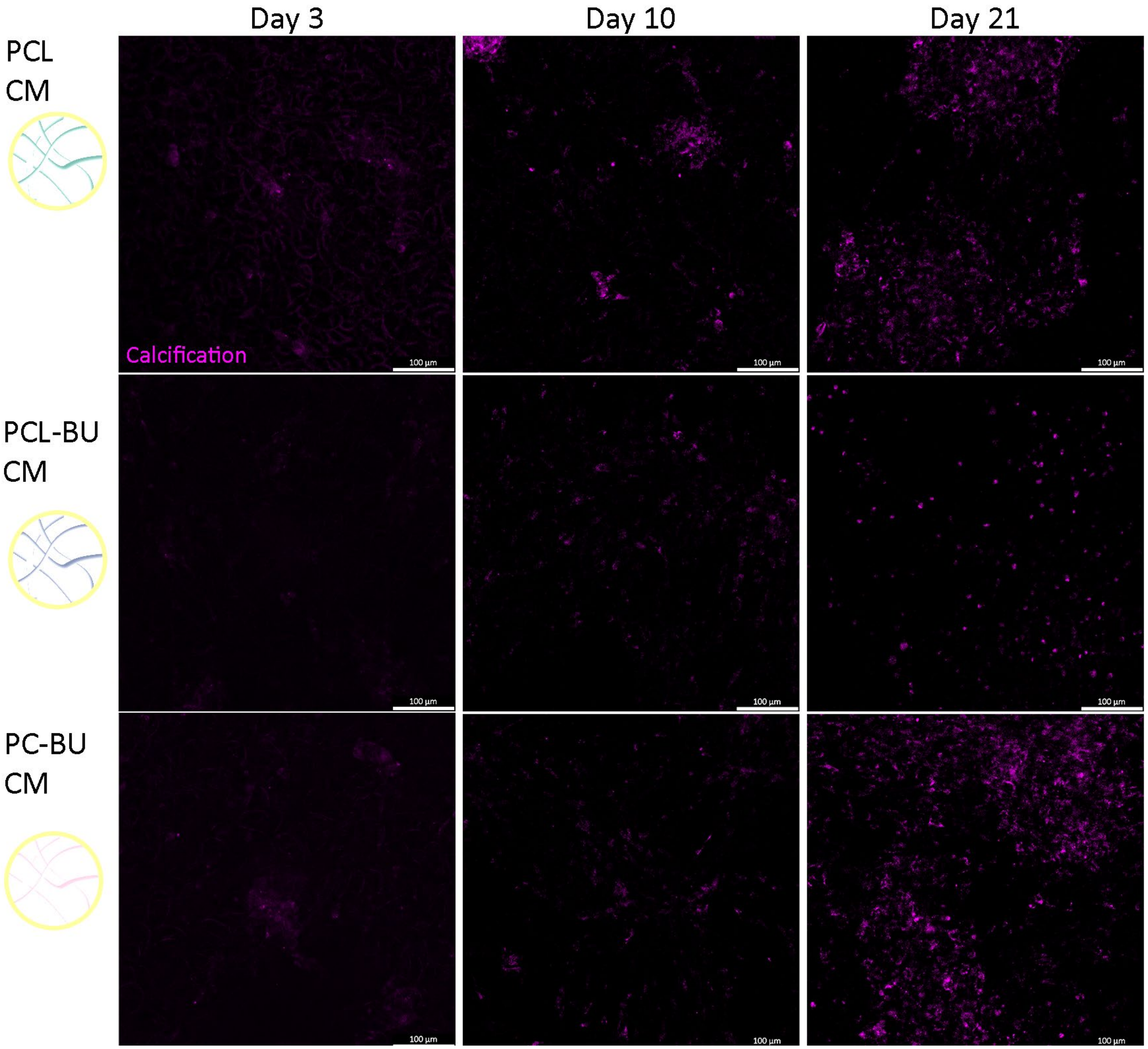

Supplementary Figure 3. Live Calcium tracing over a 21 day culture of pVICs within HVTE material constructs show an increase in calcification (pink) over time. *PCL: polycaprolactone. CM: Calcification-permitting medium. PCL-BU: Bisurea-extended polycaprolactone. PC-BU: Bisurea-extended Polycarbonate*

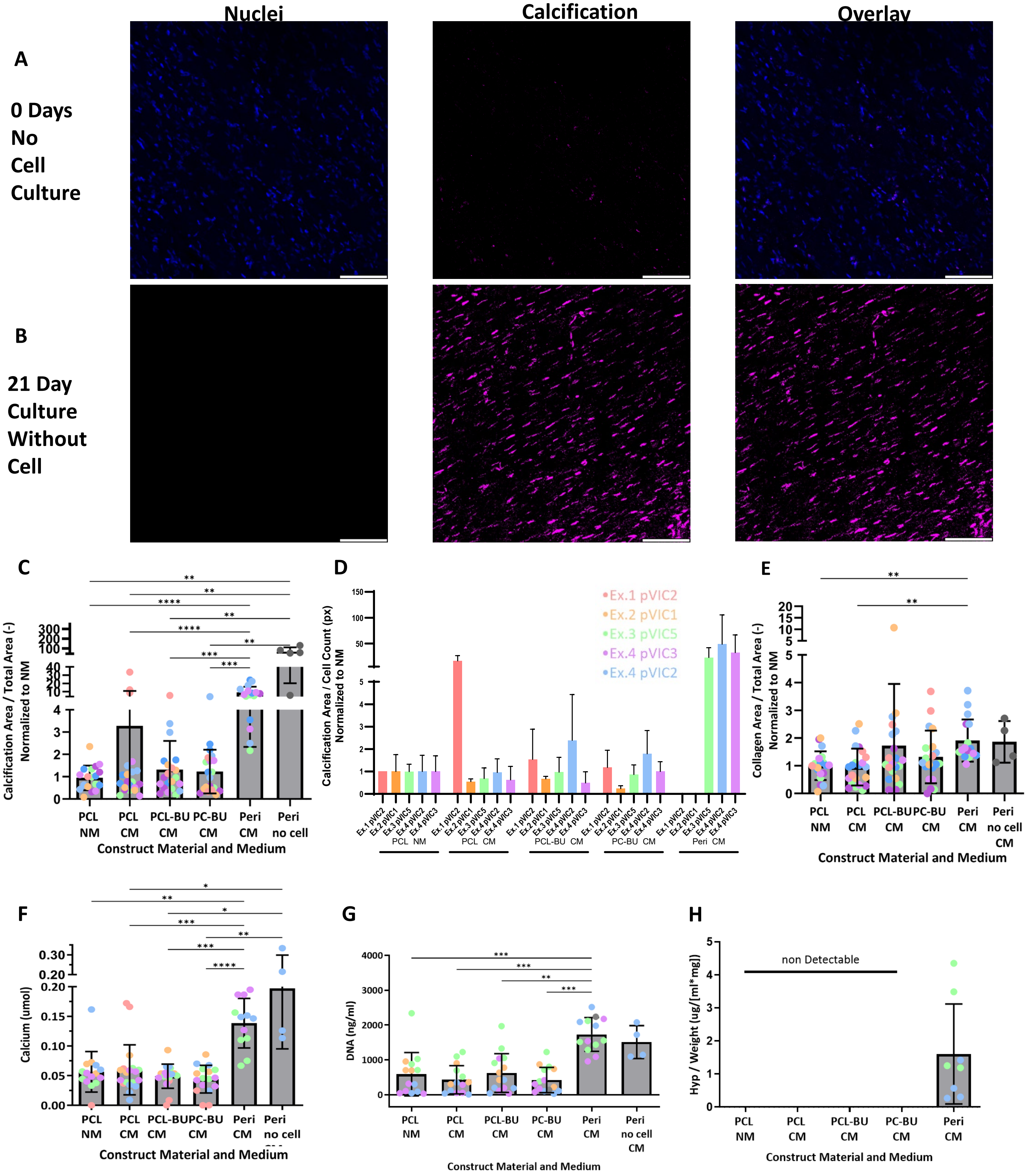

Supplementary Figure 4. Pericardial tissue culture and calcification, DNA, and Hyp assays. A. Non-cultured pericardial tissue without cell seeding showed no calcification as shown by the absence of Osteosense staining (pink). **B.** After 21 days of culture, non-cell-seeded Pericardial tissue showed a vast amount of calcification. **C.** Quantification of Calcium Area/Total Area within images showed a significant increase in calcification in pericardial tissue, independent of cell seeding. **D.** Quantification of calcium using a calcium assay within HVTE constructs compared to cellular and acellular pericardium showed an increase in calcification in pericardial tissue, independent of cell seeding. **E.** Quantification of Collagen within images showed an equal area distribution of collagen between samples. **F.** DNA assay showed a significant increase in DNA within pericardial tissue, with an almost equal amount of DNA within unseeded pericardial tissue. **G,H** Hdroxyproline (HYP) assay mediated collagen quantification showed undetectable levels of collagen within HVTE constructs, and increased levels of collagen within pericardial tissue.

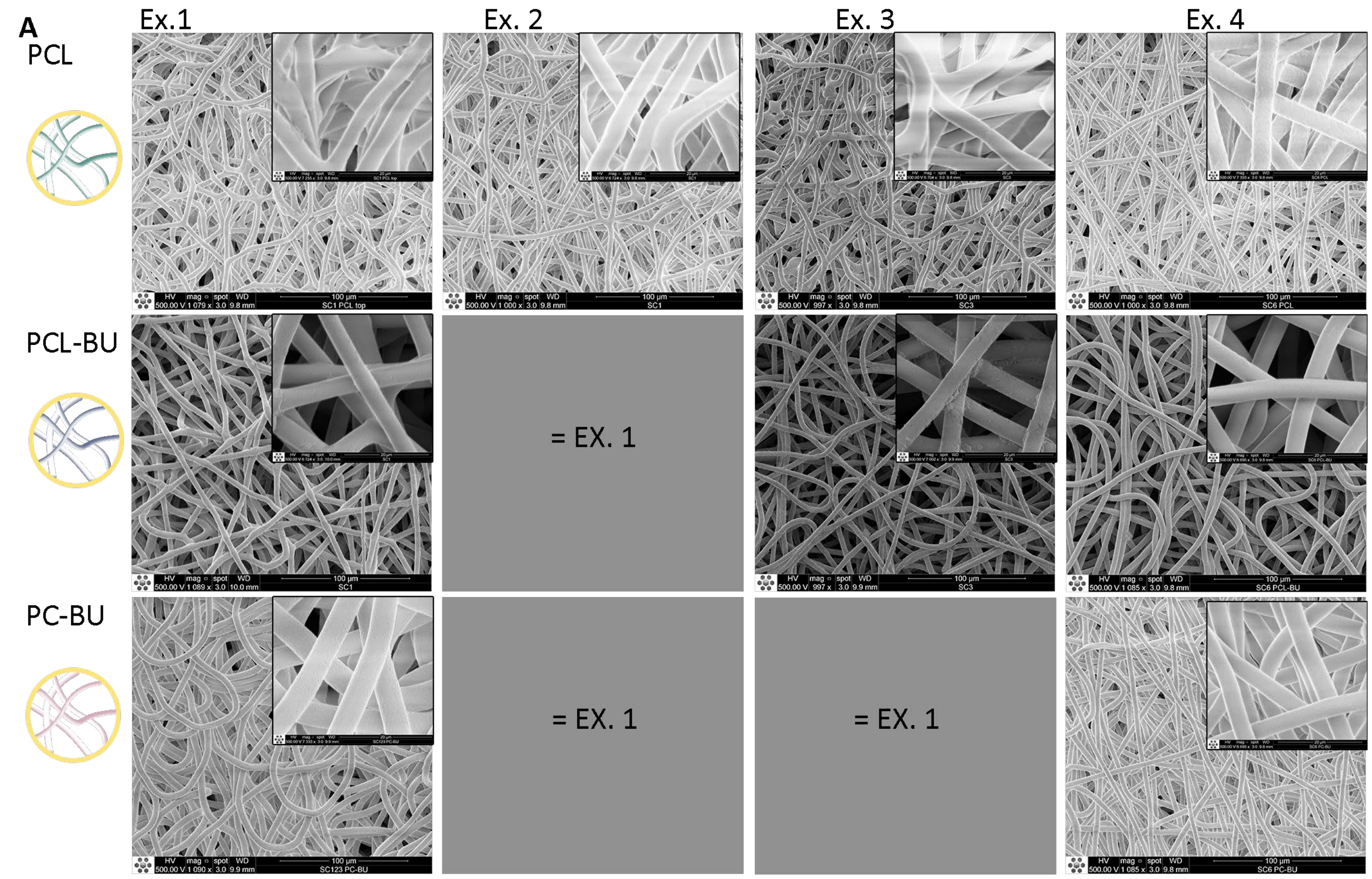

Supplementary Figure 5. SEM imaging of HVTE scaffolds over several experiments showed similar fiber morphology between experiments, with slightly bigger fibers of PC-BU material within the first 3 experiments (same Sheet of material). *=EX. 1: same material scaffolds are used for repeated experiments.*

Supplementary Table 1. Porcine VIC donor and Material Sheet per Experiment

	**Experiments from Application I compare material differences**					**Application II Strain Experiment**
	Experiment 1	Experiment 2	Experiment 3	Experiment 4a	Experiment 4b	Strain experiment
**pVIC donor**	Donor 2	Donor 1	Donor 5	Donor 2	Donor 3	Donor 2
**PCL Scaffold**	Scaffold 1	Scaffold 2	Scaffold 3	Scaffold 4	Scaffold 4	-
**PCL-BU Scaffold**	Scaffold 1	Scaffold 1	Scaffold 2	Scaffold 3	Scaffold 3	Scaffold 4
**PC-BU Scaffold**	Scaffold 1	Scaffold 1	Scaffold 1	Scaffold 2	Scaffold 2	Scaffold 2
**Pericardial Patch**	-	-	Patch 1	Patch 1	Patch 1	-

Supplementary Table 2. Scaffold Characterization Per Experiment

		**Fiber Thickness**		**Reduced Modulus**		**Molecular Weight Scaffold**			**Melting peak**		**Melting Enthalpy**	
**Experiment**	**Material**	**T (µm)**	**SD T (µm)**	**E* (MPa)**	**SD E* (MPa)**	**M_n (kg/mol)**	**M_w (kg/mol)**	***Đ***	**T_m^1 (°C)**	**T_m^2 (°C)**	**ΔH_m^1 (J/g)**	**ΔHm^2 (J/g)**
**Ex. 1**	PCL	3.99	0.39	844.1	319.4	57.6	90	1.6	54.9	NA	67.6	NA
	PCL-BU	4.32	0.25	46.4	16.6	14.8	36.8	2.5	19.9	110.7	13.7	12.4
	PC-BU	4.99	0.28	59.1	13.7	Not Measurable			13.8	146.0	3.1	9.5
**Ex. 2**	PCL	4.20	0.42	2541.4	936.7	63.8	107.1	1.7	55.3	NA	64.2	NA
	PCL-BU	is Ex. 1		is Ex. 1		is Ex. 1			is Ex. 1			
	PC-BU	is Ex. 1		is Ex. 1		Not Measurable			is Ex. 1			
**Ex. 3**	PCL	4.25	0.29	2547.5	1315.2	60.1	188	3.1	55.2	NA	68.3	NA
	PCL 2	4.25	0.47	2025.5	526.5	NA			55.2	NA	59.5	NA
	PCL-BU	4.28	0.33	32.1	6.1	16.3	34.8	2.1	Missing data?			
	PC-BU	is Ex. 1		is Ex. 1		Not Measurable			is Ex. 1			
**Ex. 4**	PCL	4.44	0.83	592.6	260.0	72.1	115.9	1.6	55.4	NA	61.1	NA
	PCL 2	4.24	0.57	NA		NA			NA			
	PCL-BU	3.97	0.17	32.2	6.1	17	34.7	2	17.2	114.1	12.8	12.4
	PCL-BU 2	4.35	0.22	27.9	7.9	24.9	61.5	2.5	16.9	112.0	10.8	9.8
	PC-BU	4.23	1.01	81.7	22.7	Not Measurable			13.4	136.9	4.7	14.1
	PC-BU 2	4.34	0.41	60.1	25.1	Not Measurable			9.2	146.2	5.6	12.8
**Experiment Average**	**PCL**	4.19	0.47	1566.2	609.2	63.4	125.3	2	55.2	NA	64.2	NA
	**PCL-BU**	4.27	0.25	38.7	11.6	18.3	42.0	2.3	19.0	111.5	13.1	12.0
	**PC-BU**	4.82	0.39	62.0	16.2	Not Measurable			13.2	144.9	3.6	10.4
